# Nanophotonic structure inverse design for switching application using deep learning

**DOI:** 10.1038/s41598-024-72125-4

**Published:** 2024-09-10

**Authors:** Ehsan Adibnia, Majid Ghadrdan, Mohammad Ali Mansouri-Birjandi

**Affiliations:** Faculty of Electrical and Computer Engineering, University of Sistan and Baluchestan (USB), PO Box 9816745563, Zahedan, Iran

**Keywords:** Computer science, Nanophotonics and plasmonics

## Abstract

Switching functionality is pivotal in advancing communication systems, serving as a paramount mechanism. Despite numerous innovations in this field, optical switch design, fabrication, and characterization have traditionally followed an iterative approach. Within this paradigm, the designer formulates an informed conjecture regarding the switch's structural configuration and subsequently resolves Maxwell's equations to ascertain its performance. Conversely, the inverse problem, which entails deriving a switch geometry to achieve a targeted electromagnetic response, continues to pose formidable challenges and necessitates substantial time and effort, particularly under the constraints of specific assumptions. In this work, we propose a deep neural network-based method to approximate the spectral transmittance of all-optical switches. The findings substantiate the efficacy of deep learning in the design of all-optical plasmonic switches, which are renowned as the fastest switches at the nanoscale. The nonlinear Kerr effect in square resonators is leveraged to demonstrate the switching performance. Juxtaposed with conventional simulations, the proposed model showcases a remarkable improvement in computational efficiency. Furthermore, deep learning can resolve nanophotonic inverse design problems without reliance on trial-and-error or empirical strategies. Compared to simulations, the mean squared error for both forward and inverse models is meager, with values of around 0.03 and 0.02, respectively. The deep learning-proposed switches exhibit excellent suitability for integration into photonic integrated circuits, substantially influencing the progression of all-optical signal processing.

## Introduction

The emergence of nanophotonics has ushered in a transformative era in optics, enabling precise control of light-matter interactions through subwavelength structures^1–3^.

This paradigm shift has catalyzed revolutionary breakthroughs, facilitating optical devices that operate beyond the diffraction limit, with wide-ranging implications for biology^2,4,5^ and nanotechnology^6–8^. The 2014 Nobel Prize in Chemistry for super-resolved fluorescence microscopy^9–11^ underscores the profound impact of these advancements in nanophotonics.

Nanophotonics harnesses optical resonances and intense localized fields produced by surface plasmons via carefully designed nanostructures^12^. Analyzing complex nanostructures often requires sophisticated numerical techniques like the finite element method (FEM)^13^ and the Finite-Difference Time-Domain (FDTD) method^14^ supported by robust solvers^15,16^. However, these methods frequently demand significant computational resources, particularly in the context of inverse design, which also relies heavily on trial-and-error approaches.

The rapid progress of artificial intelligence has propelled Deep Learning (DL) to the forefront as a transformative method for addressing existing challenges^17^. DL utilizes complex multilayer structures of Neural Networks (NNs) to extract features at multiple scales and depths, enhancing accuracy and efficiency in regression analyses^18,19^. This trend has led to a surge in DL applications across numerous disciplines^20–22^.

### Related works

Our literature review begins by studying guided-wave components before expanding to spectrum analysis in later phases. Early studies revealed the proficiency of NNs in predicting dispersion relations and photonic band gaps within two-dimensional photonic crystals^23^. Further research introduced an innovative optimization method for photonic device design utilizing an NN framework based on radial basis functions^24^. These studies employed NNs with a modest number of layers, generally ranging from two to three. Within two years, the domain of plasmonics began to harness the benefits of NNs. Investigations presented NN-based models capable of predicting the propagation characteristics of plasmonic nanostrip and coupled nanostrip transmission lines with exceptional accuracy and efficiency^25^. An innovative NN-based method markedly increased the efficiency of calculating power coupling efficiencies in photonic coupler devices^26^. Research demonstrated that a Multilayer Perceptron could predict these efficiencies in real-time, enhancing computational speed by approximately 105 times compared to the FEM.

Furthermore, the multilayer perceptron, alongside extreme learning machine NNs, has been implemented for the swift and accurate determination of dispersion relations and photonic band gaps in optimized bi- and tri-dimensional photonic crystals^27^. This strategy utilizes data from an electromagnetic solver to train and validate the NN models. Simultaneously, another research initiative focused on amplifying the quality factor, adopting a DL strategy to elevate the quality factors of two-dimensional photonic crystal nanocavities significantly^28^. This DL methodology attained quality factors exceeding those of the base cavity design tenfold and double the previously reported quality factors of 1.58 × 10^9^, achieved by optimizing air hole displacements within high-dimensional parameter spaces.

Further investigations led to the creation of an open-source deep NN model for designing polarization-insensitive subwavelength grating couplers on a silicon-on-insulator platform^29^. Additionally, a study introduced a novel design framework for integrated photonic circuit components utilizing NNs, specifically targeting strip waveguides and chirped Bragg gratings^30^. An innovative study showcased machine learning applications by employing a multilayer perceptron algorithm tailored for the efficient design of grating waveguides, mainly focusing on augmented reality applications^31^. Recently, a study showcased the application of DL for efficient spectrum prediction and inverse design of circular ring resonators (RRs), markedly enhancing computational efficiency and accuracy compared to traditional methods. However, challenges persist in data collection and the reliance on validation using the FDTD method. In spectroscopy, a DL model has been innovatively used as an optimization tool, enabling a single-peak high scattering effect in a multilayer nanoparticle structure^32^. Another seminal paper introduces a method combining convolutional NNs and recurrent NNs to extract absorption spectra from images of plasmonic structures^33^. The introduction of these innovative deep NN frameworks for spectral analysis exemplifies the transformative impact that artificial intelligence can have on optics, paving the way for novel applications and improved performance in optical communications and photonic device design^34^.

### Study limitations

The reviewed studies in this section illustrate the expansive applicability of the DL methodology across various applications^18,35^, despite facing inherent limitations and challenges^36^. Such challenges encompass the necessity for voluminous training data^37^, the reliance of prediction accuracy on the quality and representativeness of data^38^, and the intricacies associated with generalizing DL models to novel or disparate scenarios^38^. Additional considerations, including overfitting^39^, issues of model interpretability^40,41^, computational requisites^42,43^, susceptibility to adversarial attacks^44,45^, and obstacles in inverse design^46^ characterized by the many-to-one dilemma^47,48^, underscore the imperative for continual research and innovation within this domain.

### Research gap

In photonics research, the RR is universally recognized as an indispensable component for fabricating photonic integrated circuits. Despite notable advancements achieved through DL techniques in nanophotonics, a conspicuous gap persists in applying DL for spectral prediction and reverse engineering of All-Optical Plasmonic Switches (AOPS) that incorporate square RRs. This study is dedicated to investigating the capabilities of deep NN frameworks expressly devised for plasmonic resonator-based switching architectures to bridge the identified gaps in knowledge.

### Objectives and contributions

**Fig. 1:**
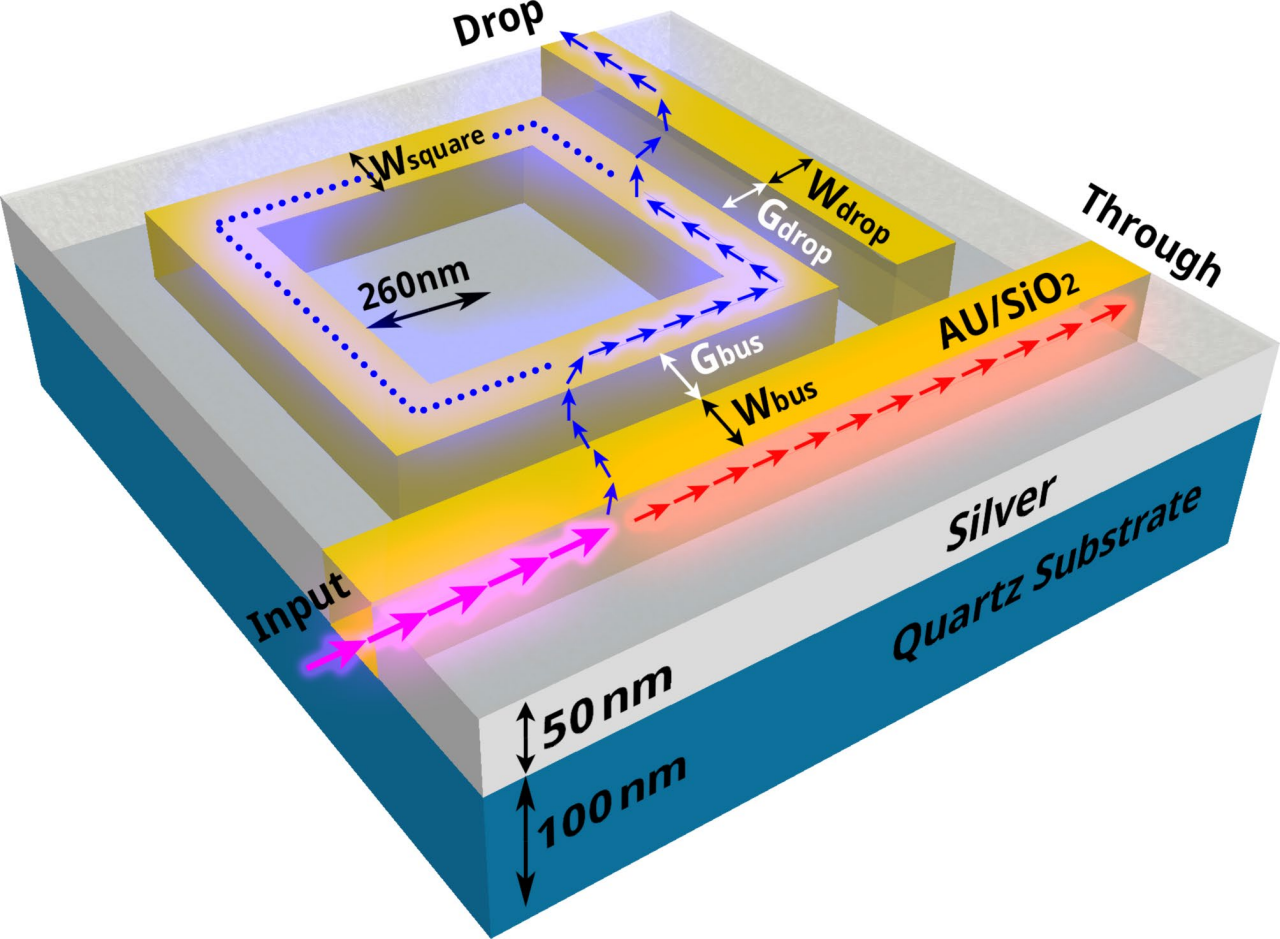
The plan of the square AOPS structure. This three-dimensional schematic presents the geometric construct and parameters used in AOPS design. The cladding layer, consisting of air, and the glass base were chosen for their favorable optical properties, which are commonly used in plasmonic device fabrication.

This manuscript centers on the escalating interest in AOPS systems founded on surface plasmon polaritons, noted for their swift response times and compact dimensions. We leverage our team's broad experience in plasmonic device research^2,49,50^ as well as in DL implementation^34^ to address the inverse design problem for square-shaped Nonlinear Plasmonic Ring Resonators (NPRR). We specifically harness the nanophotonic configuration depicted in Fig. 1, which offers a solid framework for examining the integration of DL techniques in this field. The essence of this study intersects nanophotonics with artificial intelligence, aiming to pioneer advancements in DL's ability to predict spectral behaviors and assist in the reverse engineering of AOPS configurations based on NPRRs.

Our chief goal is to introduce an innovative method incorporating DL for spectral prediction, enriching our comprehension of square resonators and facilitating the plasmonic switch inverse design process. We seek to establish a predictive model that precisely characterizes the spectral properties of these devices, employing groundbreaking methods like the Taguchi method in the data generation phase.

In this work, when we use the term "inverse design," we refer to obtaining the parameters of a specific RR design. This differs from the more comprehensive nanophotonic inverse design approaches reviewed by Molesky et al.^51^, which aim to determine the optimal structural geometry of the entire device.

In validating the effectiveness of our proposed NPRR strategy, we explore the Kerr effect^52^ and appraise switch performance using the FDTD method. This procedure involves training an NN to replicate simulations accurately, enabling precise prediction of transmission spectra and identification of resonant wavelength characteristics. While our focus primarily lies on AOPS challenges, the outlined methodology bears significant potential for addressing various issues across the nanophotonics landscape.

## Results and discussion

This section has three sub-sections. The first analyzes the forward model's findings, the second discusses its efficacy in aiding AOPS designers, and the final sub-section highlights the inverse model's capabilities. Moreover, the necessary source codes and data are publicly available under MIT license through GitHub and Zenodo repositories. These provide a comprehensive repository of coding resources essential for reproducing the simulation, establishing the model, and retrieving findings pertinent to the research’s primary concern. The repository contains MATLAB and Python scripts, detailed guidelines, code files for simulations, deep NN model training, and outcomes analysis.

### Forward model

This sub-section delves into evaluating a unique methodology designed for analyzing the transmission spectrum within an AOPS. This work utilizes a square-shaped RR rather than a circular one, which may seem counterintuitive given the general preference for avoiding sharp corners in integrated photonics due to radiative losses. However, in plasmonic waveguides, sharp bends can provide high transmission with low bending loss, owing to the strong confinement of light by surface plasmon polaritons^53^. Square-shaped resonators offer higher coupling efficiency compared to circular ones due to the extended coupling section between the bus waveguide and the resonator^54^. Moreover, plasmonic waveguides with sharp bends have been shown to maintain high transmission and low bending loss, unlike their dielectric counterparts^55^. This unique property of plasmonic structures allows us to leverage the advantages of a square geometry in our design without the drawbacks typically associated with sharp corners in conventional photonic systems.

The crux of the approach hinges on a deep NN architecture, which leverages the structural parameters of the RR as its input, as depicted in Fig. 1. The investigation broadens its scope by exploring a diverse range of waveguide widths, extending from 31 to 59 nm, and gap widths that vary from 15 to 25 nm. This comprehensive exploration created a vast training dataset encompassing 18,432 unique instances, each crafted through FDTD simulations.

The generation and preprocessing of the dataset are extensively detailed in the Methods section of the document. Additionally, applying the Taguchi method to refine the resolution of input parameters highlights the methodological rigor of this research. The specifics of the Taguchi method and the evaluation of the dataset's distribution are thoroughly discussed in Sects. S1 and S2 of the Supplementary Information. An exciting observation emerges from analyzing the computational costs and significance of structural parameters associated with varying the width and gap of the bus, drop, and square. Despite the intuitive assumption that larger widths would significantly and equally influence the resonance characteristics, the empirical evidence suggests otherwise. The gaps within the bus and drop waveguides exhibit a more pronounced impact on these characteristics, as highlighted in Figs. S2b and S3 of the Supplementary Information. This insight led to a strategic reduction in the resolution of the three abovementioned parameters to half that assigned to parameters *G*_bus_ and *G*_drop_.

The application of the Taguchi method in the data generation phase embodies a strategic approach to simulation. It ensures an optimized selection of spectra for training the NN, thus making the training process efficient and significantly effective. This method strategically explores the multi-dimensional parameter space by constructing an orthogonal array that minimizes the required number of simulations while capturing essential variations. The process involves determining discrete levels for each input parameter, designing the orthogonal array, and performing FDTD simulations for the specified configurations. Notably, this approach allowed us to reduce the required dataset to one-sixteenth of a naive parameter sweep without significantly impacting model performance. As illustrated in Fig. S3, the computational cost for each parameter is substantially reduced. The Taguchi method's efficiency in generating a representative dataset with minimal simulations represents a key contribution to our work, demonstrating how machine learning models for nanophotonic design can be trained more effectively and with lower computational overhead. The specified range of parameters empowers the NN to make precise predictions regarding the spectral attributes of millions of RR structures, thereby showcasing the efficacy and robustness of the proposed approach in spectrum prediction.

**Fig. 2:**
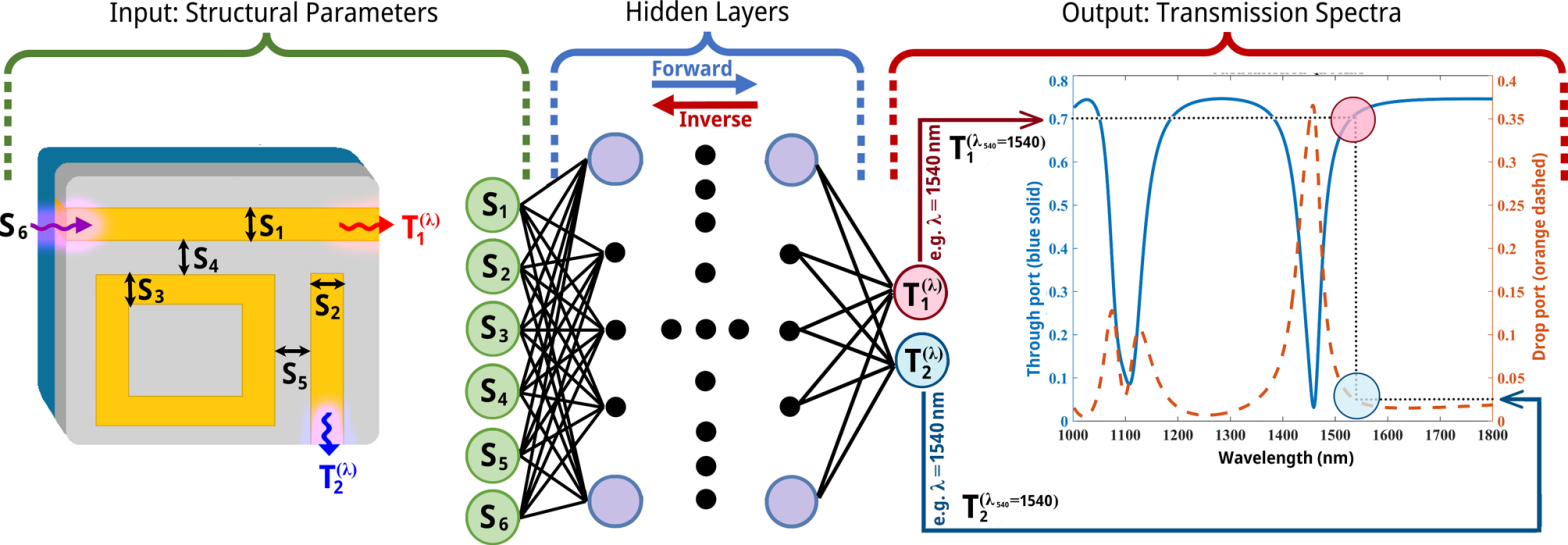
The schematic of the deep NN architecture. The prediction of the spectrum of the AOPS utilizes this deep NN architecture, which takes inputs such as the geometrical specifics (like waveguide width and waveguide gap) and the wavelength of the input light. After traversing across eleven hidden layers, the NN predicts the transmission over different wavelengths, indicating the entire spectrum achieved from the AOPS device. This NN makes it possible to identify sophisticated connections between input parameters and the resulting transmission spectrum, giving a deeper understanding of the AOPS's behavior and performance.

After this phase, our team embarked on the NN training utilizing a dataset crafted with meticulous precision. The methodology underlying the NN’s architecture, which leverages input variables to predict the spectral response relevant to the designated AOPS, is unveiled in Fig. 2. This figure also showcases the NN’s output, namely the transmission spectrum, across a 1000–1800 nm wavelength range. The architecture chosen for this endeavor relies on a fully connected, layer-based NN, incorporating 11 optimized hidden layers and 160 neurons within the central hidden layer, resulting in 34,612 parameters. A detailed rationale for selecting this particular configuration is articulated in Sect. S3 of the Supplementary Information.

**Fig. 3:**
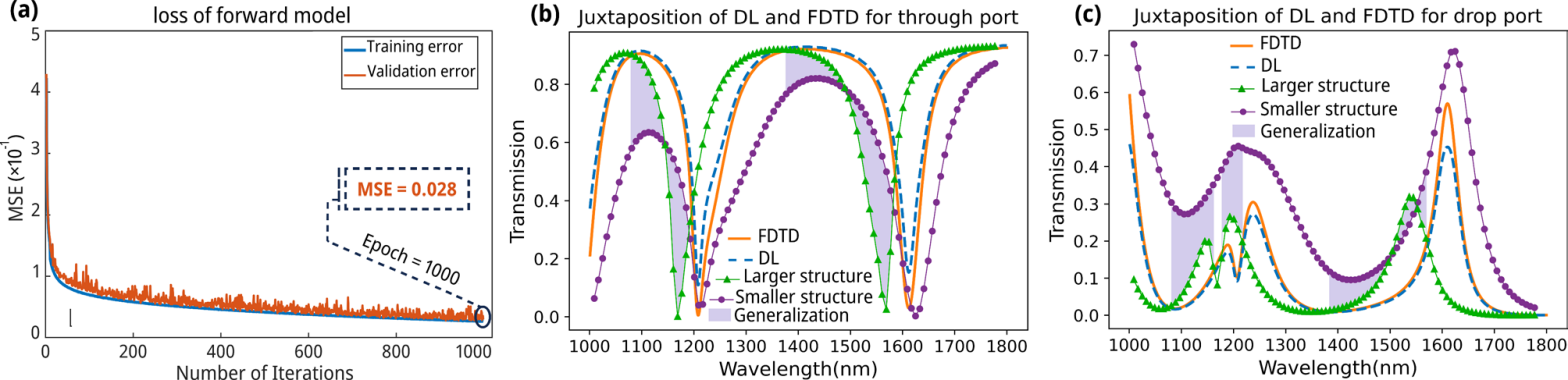
The NN’s performance in estimating the spectrum. (**a**) The training loss, which shows significant drops, hints at the NN’s ability to identify trends in the data. (**b**) A juxtaposition of the approximated values of the NN and the actual spectrum in the through port and also the nearest training instances. (**c**) The corresponding comparison of the NN’s predicted spectrum and the actual spectrum in the drop port. The gray area highlights the NN’s generalization ability.

After completing the training phase, we archived the NN's weights, enabling their straightforward retrieval and application. We conducted an in-depth analysis to shed light on the practical application of the DL methodology in accurately predicting the transmission spectrum. The training loss graph, depicted in Fig. 3a, elucidates the NN’s performance throughout the training process, revealing the network’s proficiency in accurately predicting transmission spectra, evidenced by a minimal validation loss value of 0.028. To evaluate the model's generalization capabilities, we calculated the loss on a held-out test dataset comprising 15% of the total data. The forward model's test loss was 0.03, which is closely aligned with the validation loss. This consistency between test and validation performance indicates that our model generalizes well to unseen data and is not overfitting. Subsequently, we redirected our attention to evaluating the network's capability to simulate spectra not encompassed within the training dataset. The network’s comprehensive adaptability was further assessed by comparing the predicted transmission and actual spectra, as illustrated in Fig. 3b,c.

This comparison engenders a thorough investigation into the similarities and differences between the spectra. Notably, the spectral prediction derived from the forward model closely mirrors the actual spectra, demonstrating the network’s exceptional ability to accurately match spectra for parameter values not present in the training set, thereby highlighting its potential to discern and replicate features not present in the initial training data. This observation is corroborated by plotting proximate samples from the training set, as shown in Fig. 3b,c, where the network transcends simple interpolation or averaging of the nearest training spectra (refer to the gray area in Fig. 3b,c). Instead, it exemplifies the network’s capacity for generalization and identifying novel, previously unrecognized features, thereby indicating that the NN does not simply conform to the data but actively explores substantial patterns and configurations within the input and output data. The spectra plotted for the larger and smaller structures are the simulated spectra. It is important to note that the spectra presented in Fig. 3b,c (as well as Fig. 6b) were not chosen randomly or intentionally for favorable results. Instead, these examples were deliberately chosen to represent the most challenging cases from the extremes of our training distribution. This approach allows us to evaluate our model's performance under worst-case scenarios. By showcasing these challenging examples, we aim to provide a more stringent and transparent assessment of our model's robustness and generalization ability, rather than presenting only the most favorable outcomes.

As seen in Fig. 3b,c, the model's performance exhibits slightly lower accuracy in the resonant region compared to other spectral areas. This phenomenon, also observed in our previous work, can be attributed to an inherent imbalance in the training data. Each structure typically has a single dominant resonant frequency, resulting in other data points representing non-resonant cases for every single data point corresponding to the resonant wavelength. This significant disparity in representation poses a challenge for the model to accurately learn the characteristics of the critical resonant region. As illustrated in Fig. S6 of the Supplementary Information, increasing the complexity of the NN improves the model's accuracy in predicting the resonant wavelength. However, we acknowledge that there is still room for enhancement, particularly in these challenging spectral regions.

### AOPS design based on the deep NN

While the NN training does require extensive data from numerical simulations, our DL-based approach can subsequently circumvent the necessity for extended numerical method computations in the analytical process. This substantially expedites the design and optimization of the nanophotonic structures after the initial model training is complete. (refer to Fig. S9). Our methodology facilitates incorporating various waveguide characteristics into the trained NN, producing outcomes within minutes. This efficiency grants us access to a vast repository of spectral responses for a wide array of structures, enabling the rapid retrieval of needed spectral data by navigating through this collection.

Our deep NN model was trained exclusively on low-intensity simulations, allowing efficient prediction of the nanophotonic structures' linear, low-intensity transmission spectra. The model is used to optimize structural parameters for a distinct peak in the drop port spectrum at a specific target wavelength, forming the basis of our AOPS design. We then employ FDTD simulations to evaluate the nonlinear, high-intensity behavior of the optimized structure. This two-step process enables efficient design and validation of the AOPS, leveraging the computational advantages of machine learning while accurately capturing the full range of optical phenomena involved in the switching mechanism.

The choice of the most suitable switching mechanism may depend on the specific design goals of the optical switch. DL offers designers unprecedented flexibility, with its capability to generate a broad spectrum of spectra in a shortened timeframe. This flexibility allows designers to make well-informed decisions about the most appropriate structure for various applications, enhancing confidence. This part of our research mainly focuses on the observable differences between the through port and the drop port across the spectra produced by the NN, highlighting the switching features of the RR structure. The emphasis on the third telecommunications window is due to its lower optical attenuation compared to shorter wavelengths, substantially benefiting optical communication systems^56^. Moreover, adopting a high contrast ratio is deemed beneficial, as it increases the system’s resilience to noise and improves error detection capabilities in the operational deployment of switching devices. This advantage, in turn, enhances the operational reliability of these devices, marking a notable advancement in the field of optical communications^57^.

**Fig. 4:**
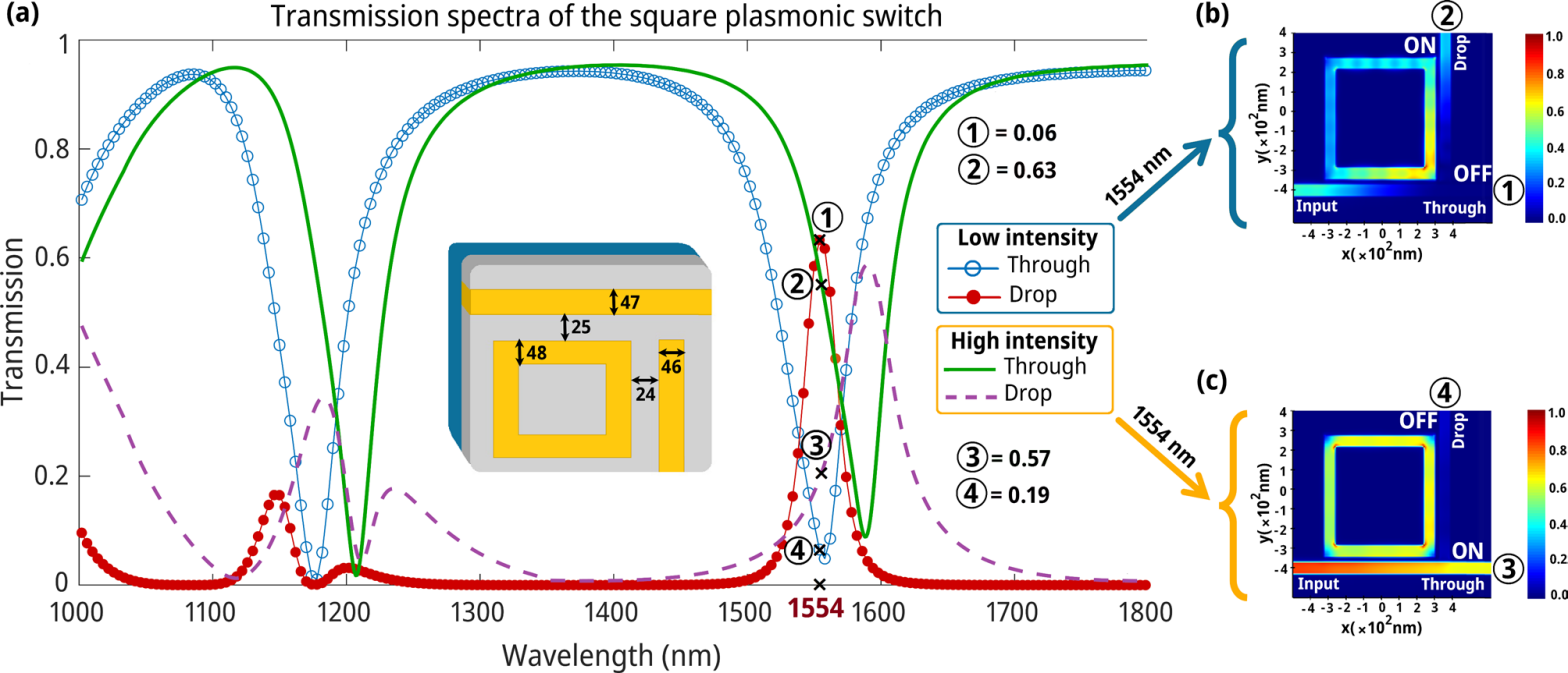
The performance of the switching mechanism. (**a**) The transmission spectra of the square NPRR. The resonant wavelength is notably occurring at 1554 nm. With an increase in the input light’s intensity, a spectral redshift occurs in the resonant wavelength due to nonlinear effects. (**b**) The low-intensity optical field of the AOPS. Around 63% of the incoming light goes through the drop port, while 6% goes through the through port, signifying an "ON" situation for the drop port and an "OFF" situation for the through port. (**c**) In an AOPS under high intensity, around 57% of the input light passes through the through port, while about 19% traverses via the drop port, indicating an "ON" state and an "OFF" state, respectively.

Upon determining the optimal structural parameters through DL, we performed FDTD electromagnetic simulations to minimize errors associated with the DL methodology and accurately ascertain the transmission spectra of the structure. The essential design parameters for a square NPRR and its transmission spectrum are prominently featured in Fig. 4a. Investigating the switch’s linear and nonlinear domains sheds light on its operational mechanisms. The electromagnetic simulation of the switching operation under both low and high optical intensities is vividly illustrated in Fig. 4b,c, enhancing our conceptual understanding of the process.

Optical power is effectively coupled to the RRs within the linear operation domain when low-intensity light is introduced at the resonant wavelength, enabling transmission at the drop port (see points ① and ④ in Fig. 4). This phenomenon occurs when the resonant wavelength coincides with the linear resonant condition of the RRs. In contrast, an increase in light intensity triggers the onset of the nonlinear Kerr effect, characterized by the change in a material’s refractive index in response to the intensity of the light (see points ② and ③ in Fig. 4). The transmission spectra reveal a redshift due to the Kerr effect, causing the light to diverge from the RRs’ resonance, thus reducing power coupling efficiency. Consequently, rather than being coupled to the RRs and directed to the drop port, the light proceeds to the through port. The efficiency of light coupling to the RRs diminishes due to the resonance condition shifting away from the initial wavelength, primarily because of the redshift induced by the Kerr nonlinearity. This dynamic illustrates the Kerr nonlinear effect’s notable influence on the structure’s transmission characteristics. By modulating the light intensity, we can precisely control the resonance conditions and the power coupling to the RRs, thereby enabling enhanced manipulation of light transmission between the device's ports. Consequently, the Kerr effect modulates light transmission within this nonlinear photonic structure, offering a nuanced control mechanism for optical switching applications.

To provide a more vivid depiction of the AOPS mechanism, we performed electromagnetic simulations using input light pulses of variable intensities on the square geometry at the resonant wavelength of 1554 nm. The optical field patterns computed under low-intensity linear and high-intensity nonlinear excitation conditions are graphically represented in Fig. 4b,c, respectively. With minimal input power, the field profile strongly indicates a robust resonant coupling of the signal into the square ring, consistent with signal propagation towards the output waveguide, as anticipated within the linear regime’s operational principles (Fig. 4b). However, as the input intensity increases, the transmission spectrum shifts towards the left, attributable to the refractive index modification induced by the Kerr effect.

Consequently, the pronounced field pattern shown in Fig. 4c demonstrates negligible coupling into the square ring, with the pulse primarily proceeding undisturbed through the input waveguide, corroborating the theoretical principles of nonlinear switching activation. The threshold power in Fig. 4 is 9.6 MW/cm^2^.

Figure 4 compellingly demonstrates a notable correlation between the optical field trends and the operational functionality of the AOPS in both its "OFF" and "ON" states. This alignment underscores the intricate interplay between input light intensity and the resultant optical field distribution within the square ring structure, highlighting the pivotal role of the Kerr effect in modulating the device’s transmission characteristics. These simulations elucidate the AOPS's dual operational states, offering insights into the mechanism's efficacy in switching between transmission modes. Switching between transmission modes effectively affirms such structures' technological viability and adaptability in advanced optical switching applications. Enhancing contrast in the transmission spectrum is advantageous; however, adopting a strategy that promotes a more pronounced decrease in the transmission spectrum can also effectively select the optimal spectrum^56,58^. The inherent characteristics of the switching mechanism fundamentally inform the selection of a transmission spectrum with a sharper decline^59^. Identifying a spectral dip with a steeper incline enables a more explicit and quicker transition during the switching operation. Detailed insights into our strategy for selecting a transmission spectrum that meets this criterion are provided in Sect. S4 of the Supplementary Information. Initially, we establish the connection between a substantial reduction in the transmission spectrum and its second derivative, as shown in Fig. S7. We then apply this relationship to the dataset generated using the DL methodology, explicitly targeting the wavelength of 1310 nm. This particular wavelength is selected as it falls within the second telecommunications transmission window, chosen for its minimal chromatic dispersion within this bandwidth^60,61^.

**Fig. 5:**
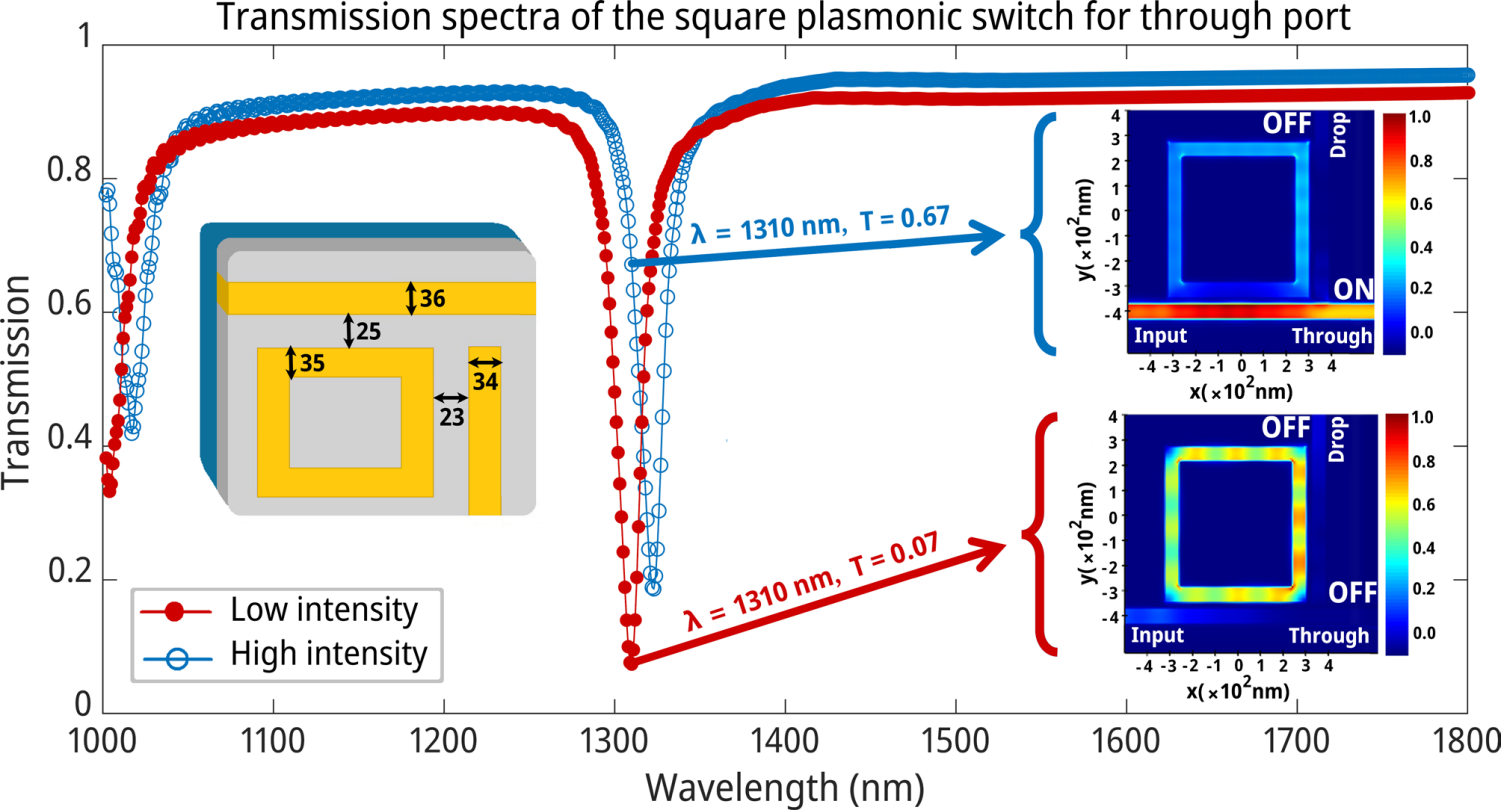
The efficiency of the switch structure. The square NPRR switch's transmission spectrum and the AOPS's optical field were analyzed in low and high-intensity states. The switch's performance was evaluated by examining its optimized transmission spectra, revealing a resonant wavelength at 1310 nm. Increasing the input light's intensity caused a redshift in the resonant wavelength due to nonlinear effects. The AOPS's light field was also depicted. At low intensity, around 7% of the incoming light passed through the port, indicating an "OFF" state. Approximately 67% of the input light passed through the through port at high intensity, indicating an "ON" status. The drop port remained "OFF" in both cases with minimal transmission.

Figure 5 displays the transmission spectra for the proposed plasmonic switching device under conditions of low and high optical input intensities. At lower intensities, where linear optical effects dominate, a distinct extinction dip is visible in the transmission spectrum at the through port, near the resonant wavelength of the plasmonic square ring (red closed circles in Fig. 5). This sharp dip indicates a strong coupling of the incident light with the resonant square ring, consistent with the switch’s theoretical model in the "ON" state. However, as the intensity increases where nonlinear effects prevail, a spectral redshift in the extinction dip occurs (blue opened circles in Fig. 5). Due to this resonance shift, the incident light becomes out of sync with the original resonant coupling condition, leading to reduced power transmission to the square ring and a transition to the "OFF" state, as depicted by the transmission profile.

The simulation results bolster the conceptual model, demonstrating how the modulation of resonance properties dependent on intensity can enable optical switching capabilities in the designed plasmonic nanostructure. This approach validates the theoretical underpinnings of the switch’s operation and underscores the practical feasibility of employing intensity-dependent resonance modulation as a mechanism for optical switching within plasmonic nanostructures.

These examples illustrate the notable potential of our proposed DL methodology for identifying an optimal configuration for the plasmonic switching device. Moreover, we demonstrated the forward model's utility in optimization efforts, showcasing its value in refining design parameters. Investigating the proposed plasmonic switching device utilizing the forward deep NN revealed insightful revelations regarding its operational dynamics and design considerations. The ability of the trained NN to obviate the need for extensive computations of numerical methods highlights its practical utility in streamlining the design process. The exploration of optimal geometric parameters, guided by the network’s predictions, further accentuates the efficacy and precision augmented by DL in designing AOPS. The groundwork established in this discussion lays a robust foundation for comprehending and utilizing the forward model, facilitating its implementation in real-world applications. As we progress to the following sub-section, our attention shifts toward the real-world application and the experimental validation of predictions made by the inverse deep NN. This transition sets the stage for a deeper understanding and applying the forward model in practical settings.

### Inverse deep NN and inverse design

Our research illuminates the profound efficacy of DL in adeptly navigating the complexities of inverse design challenges, a pivotal domain straddling engineering and physics disciplines. The inverse design involves identifying a desired spectral output and subsequently deducing the precise geometry capable of reproducing this output with high fidelity. Our studies highlight the remarkable success of NNs in achieving this goal, demonstrating their capacity to resolve inverse design challenges with notable precision.

At the heart of our methodology lies the strategy of selecting a random target spectrum as a benchmark, upon which the trained network is tasked with inferring the input variables necessary to engender a spectrum that closely mirrors the target. This approach facilitates the precise determination of input parameters essential for attaining the desired spectral outcome, enhancing the efficiency of the inverse design process. To ascertain the physical validity of these spectra, we derived the target spectrum from a feasible configuration within the AOPS and ensured that the spectrum originates from a physically plausible structure. Such a strategy bolsters the network’s proficiency in predicting input parameters that yield corresponding spectra, adhering to real-world constraints^62^, and ensuring the applicability of the designs in practical scenarios. This methodology showcases the potential of DL in transforming the landscape of inverse design and underscores its utility in streamlining the design process, thereby facilitating the development of innovative solutions within engineering and physics.

**Fig. 6:**
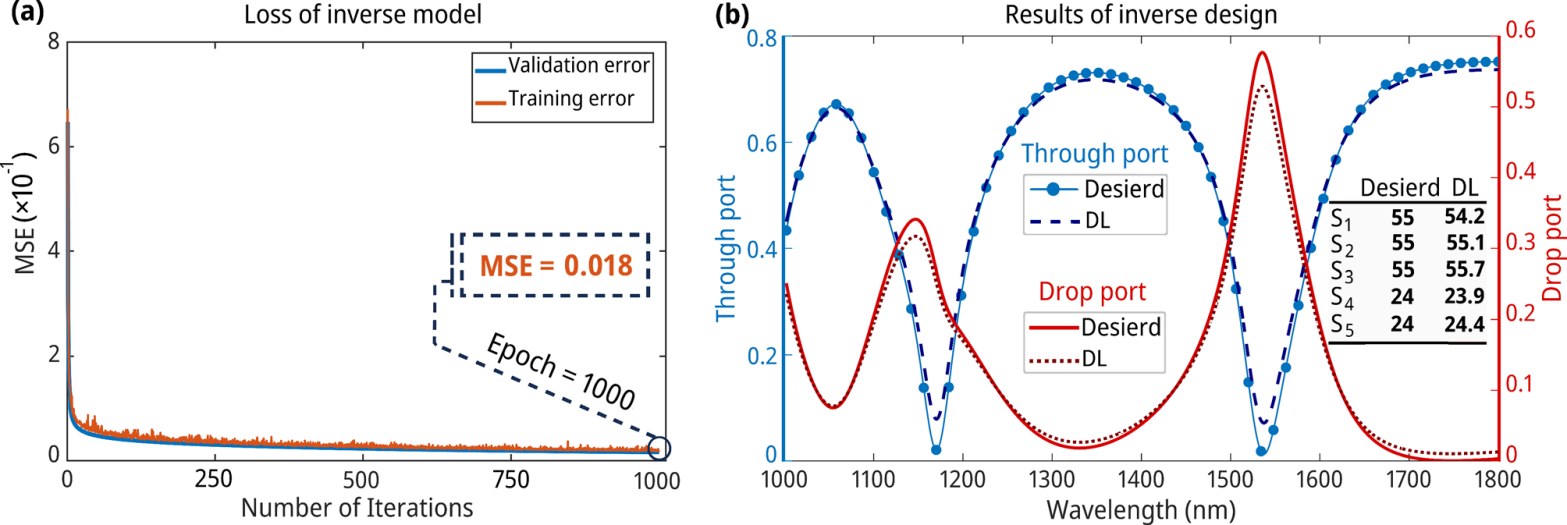
The outcomes of inverse design for the AOPS using the DL approach. (**a**) The illustration of training loss shows a remarkable decrease over initial epochs. The low loss value confirms that the NN successfully learns and recognizes patterns in the data throughout the training process. (**b**) The capability of the inverse model to predict design parameters for the transmission spectrum in the furthest data point away from the training dataset. The miniature table serves as an identifier of the design parameters used in the investigation.

We deliberately chose a structure substantially different from those used in the network’s training to ensure the accuracy and reliability of the model. This process confirms the model's ability to predict the desired spectrum, even for unseen configurations precisely. Figure 6a shows the inverse model’s performance throughout training, indicating the network’s precision in predicting geometric details with a minimal validation loss value of 0.018 and test loss value of 0.019. Additionally, Fig. 5b compares the transmission spectra of the targeted AOPS configuration with the predicted transmission spectrum derived via the DL methodology. The minimal differences between these spectra highlight the DL method’s proficiency in effectively predicting the desired transmission spectrum.

Highlighting the instrumental role of the FDTD method in deriving the spectra depicted in Fig. 5b is imperative to address the inverse problem. This approach mitigates potential inaccuracies associated with the forward model. Our inverse model’s comprehensive training and validation enhance its credibility and demonstrate its prowess in predicting intricate structural designs to generate the anticipated spectra. Our methodology simplifies the resolution of inverse design dilemmas, circumventing the need for labor-intensive manual derivation and computation of inverse equations. While simpler prediction methods like nearest neighbor interpolation or linear regression might suffice for less complex systems, the intricate, nonlinear relationships inherent in nanophotonic structures necessitate more sophisticated approaches. Our DL model excels in capturing the complex interactions between multiple geometric parameters and their influence on spectral responses, particularly in high-dimensional parameter spaces like square resonators. The NN's multilayered architecture enables it to learn hierarchical representations of the data, effectively modeling the nonlinear dependencies that simpler methods struggle to capture. This ability becomes increasingly valuable as the parameter space expands, offering superior predictive power and generalization capabilities compared to traditional interpolation or regression techniques. Our model's performance in predicting spectra for configurations at the extremes of the parameter space further demonstrates its robustness and utility in navigating the complex landscape of nanophotonic design.

To enable a comprehensive comparative analysis of recent advancements in the field, a summary of key photonic research studies is provided in Table 1. This table includes details such as the type of structure, the number of input parameters, the configuration of hidden layers, and the model accuracy as measured by various evaluation metrics. Directly comparing the results of different DL methods can be challenging due to the diversity in structures, variations in NN architectures, and the use of different evaluation metrics. For example, the table presents the performance of the models in terms of Mean Squared Error (MSE), Mean Percentage Absolute Error (MAPE), Mean Relative Error (MRE), and Mean Squared Logarithmic Error (MSLE), which are among the most commonly used evaluation metrics in this domain.

**Table 1:** A comparative analysis of applying DL in the design of photonic structures.

Article	Structure	Number of input parameters	Hidden layers and their neurons	Error	Sample size
Verma et al.^63^	Nano‑structured dimers	3 (all geometry parameters)	50–50–50–50–50	MSE < 0.05	50,000
Peurifoy et al.^32^*	Nanophotonic particle (7 structures)	9 (all geometry parameters)	250–250–250–250	MRE = 1.5%	50,000
Singh et al.^64^	Meta‑structures	8 (7 geometry parameters and 1 wavelength)	1024–512	MSE = 0.012	4000
Zhang et al.^65^*	Plasmonic waveguide (3 structures)	15 (all geometry parameters)	200–300–300–300–200–15	accuracies > 90%	20,000
Singh et al.^66^	1D photonic crystal	8 (4 geometry and 4 material parameters)	4–8–16–32–55	MSE = 0.014	50,000
Malkiel et al.^67^	Plasmonic nanostructure	8 (all geometry parameters)	3 parallel group layers with 8 Join-layers depth	MSE = 0.0582	> 15,000
Adibnia et al.^34^	Plasmonic nanostructure (circular resonator)	6 (5 geometry parameters and 1 wavelength)	60–60–60–60–60–60	MSLE < 4 × 10^–4^	147,456
Chen et al.^31^*	Grating waveguides (6 structures)	5 (3 geometry and 2 material parameters)	five to six hidden layers of tens to hundreds of neurons	MAPE < 2%	3000–14,000
Li et al.^68^	Plasmonic nanostructure (periodic gold nanodisks)	3 (all geometry parameters)	320–320–320–320	Relative error = 97.5%	2254
Wu et al.^69^	Plasmonic antennas	3 (all geometry parameters)	300–450–450–450–450	MSE = 0.478	3024
This work	Nonlinear plasmonic square resonator	6 (5 geometry parameters and 1 wavelength)	5–10–20–40–80–160–80–40–20–10–5	MSE < 0.03	$\frac{\text{18,432}}{16}=1152$

*The information relates to the most complex structure investigated in the study.

As seen in the comparative table, nanophotonic structures like plasmonic structures and cases with an increased number of input parameters require more complex NN architectures. Consequently, this leads to higher computational costs. Nonetheless, our work, when compared to similar studies, has achieved relatively desirable error rates with a less complex architecture. As seen in this table, the application of the Taguchi method, which significantly reduces the amount of data required for training the NN, distinguishes our work from other studies.

As we dissect our findings, critically examining our methodologies is essential, recognizing the inherent limitations. The deep NN's success relies on the quality and diversity of the training data. Although utilizing FDTD simulations to generate datasets introduces a degree of bias, it is essential to acknowledge that data acquisition is a notable challenge in implementing DL strategies. Understanding the rationale behind the model's decisions presents a challenge, prompting further investigation into model explainability in the nanophotonic domain. As our study concludes, we hope that the insights provided will inspire specialists in the field to conduct more in-depth investigations. The Inverse design by deep NN stands out not only as a time-efficient approach in nanophotonic design but also as a catalyst for exploring new possibilities in optical communication.

## Conclusion

This study successfully employed DL techniques to establish a notable correlation between the spectroscopic attributes and the operational performance of plasmonic square RR. By harnessing the power of DL, our research overcomes the intricacies of inverse design, thus enhancing the functionality of AOPS based on NPRR. Moreover, this study demonstrated that the Taguchi robust design is a potent tool that can improve the quality of datasets, minimize data generation expenses, and yield substantial advantages in the data generation phase. The architecture of our NN features 11 overcomplete hidden layers, with the central layer comprising 160 neurons, and the training process extended over 1000 epochs. These parameters were carefully adjusted to achieve an ideal equilibrium between swift convergence and accurate prediction of spectral characteristics for AOPS’s spatial configurations. It is crucial to emphasize that these parameters can be adapted depending on the unique challenges encountered. In situations marked by uncertain outcomes, cautiously adjusting these parameters is recommended to maintain the DL model’s reliability and safety. The transmission spectra predicted by NNs display exceptional agreement with those obtained through FDTD simulations, highlighting the unparalleled accuracy of our DL approach. This methodology substantially reduces computational costs compared to traditional numerical solvers, offering rapid and economical spectral predictions for RR arrangements.

Furthermore, our approach tackles the challenge of inverse design, creating optimal geometries for the desired optical response spectra. Our DL model is validated using physically plausible configurations, confirming the feasibility of the proposed switches for incorporation into photonic integrated circuits. Our findings have practical relevance and demonstrate their applicability in various real-world scenarios.

The fusion of nanotechnology’s precision and artificial intelligence’s computational might and pattern recognition capabilities heralds a new era of scientific innovation. This synergy is set to drive notable breakthroughs and foster the development of pioneering applications, marking a transformative shift in scientific exploration and the potential to revolutionize various fields. As this paper concludes, we invite readers to review our accomplishments in Animation S1.

## Materials and methods

This section describes the methods used in our investigation to ensure the results can be replicated. This section expounds on the methodologies utilized in this study, commencing with a concise overview of the theoretical underpinnings. Further comprehensive elucidation of these foundations is available in the Supplementary Information S1. Considering the multifaceted nature of this study, the theoretical framework was split into two distinct categories within Sects. S5 and S6. The initial sub-section delves into the mathematical equations foundational to the design and modeling of AOPS, while the subsequent portion is dedicated to elucidating the formulation of deep NN strategies. Within the defined mathematical framework (Eq. S4), we adeptly integrated the wavelength of the incident light into the forward model of the NN. This integration endowed the model with the remarkable capability to accurately predict transmittance values across discrete and broadband wavelengths, inviting the reader into a realm where precision and practicality meet innovation. Following this examination of the theoretical foundations, the upcoming sub-section will shed light on generating and preparing data. The final sub-section will comprehensively outline the steps followed when training the NN.

### Generation and preprocessing of data

The study evaluates the efficacy of AOPS by selecting and adjusting specific design parameters such as the widths (*W*_bus_, *W*_drop_, and *W*_square_) and gaps (*G*_bus_ and *G*_drop_) of the waveguides. Changes range from 31 to 59 nm for widths and 15 to 25 nm for gaps to optimize performance. The training dataset, generated through FDTD simulations, is accessible under an MIT license on GitHub and Zenodo, with detailed composition outlined in the Supplementary Information (Sect. S2). The dataset's construction using FEM or FDTD is time-consuming, with significant computational demands discussed in Sect. S7. To enhance the NN's performance, we adopted the Taguchi method, supported by software solvers like Qualitek-4^70^ and Minitab^71^ (detailed in Sect. S1) to prioritize and select parameter resolutions for data generation, significantly involving statistical analysis to assess parameter influences^72^. Despite the computational intensity, this method produced a comprehensive dataset in about a month using three 3.1 GHz 16-core computers, training the NN to predict vast datasets swiftly, as opposed to the more extended data generation phase.

### Procedure of training

This research utilizes the Keras library^73^, which has been integrated into Google’s TensorFlow^74^ since 2016, to execute tasks in Python^75^ within the Anaconda^76^ environment. We assessed multiple machine learning packages, leveraging Pandas^77^ for data preprocessing and Scikit-learn^78^ for model training. NumPy^79^ was crucial for handling matrices in developing the regression model. All relevant codes are available on the GitHub repository.

The study features an NN with an overcomplete hidden layer whose dimensionality matches or exceeds the input space. Following the formula ${2}^{\left({N}_{L}-1\right)/2}$, we adopted an approach to determine the neuron count in the central hidden layer based on the total number of hidden layers (*N*_L_). We optimized the NN architecture for maximum neurons centrally, with neuron counts halving towards the input and output layers. This architecture includes an odd number of hidden layers to maintain symmetry.

Using the Fibonacci sequence to adjust the central layer's neuron count, we explored different neuron scaling strategies. The dataset was divided into 70% training, 15% validation, and 15% testing, with a batch size of 80 and updates based on training loss-derived gradients. It is crucial to emphasize that our data division methodology was conducted on a simulation-by-simulation basis, not by individual data points. Details on hyperparameter tuning, including layer counts and training epochs, are discussed in the Supplementary Information (Sects. S3 and S7), which also addresses computational cost implications.

The study deviates from conventional activation functions like ReLU, opting for Leaky ReLU^80^ to prevent gradient vanishing and enhance optimization convergence. This function's suitability for continuous value estimation tasks is highlighted, fitting well with the study's objectives. The following equation deliveries the Leaky ReLU function’s formula^34^:

1 $$\text{Leaky ReLU}=\left\{\begin{array}{cc}\alpha x& x\le 0\\ x& x>0\end{array}\right.$$

In this equation, *x* is the input value, and *α* is a small positive constant that sets the function’s slope for negative inputs, set at 0.2 during training. For error estimation, we compared the anticipated spectral output of the proposed deep NN with the actual spectral values using MSE. The calculation of MSE follows this equation^81^:

2 $$\text{MSE}=\frac{1}{n}\sum_{i=1}^{n}{\left({y}_{\text{pred}}-{y}_{\text{true}}\right)}^{2}$$

where *n* denotes the total number of data points, *y*_pred_ is the predicted value, and *y*_true_ represents the actual value computed utilizing the FDTD technique. The Adam optimizer, known for its fast convergence compared to stochastic gradient descent, was chosen for its ability to handle nonlinear datasets and adaptively adjust learning rates for each parameter, optimizing memory use^82,83^. This optimizer was integral in the iterative refinement of the deep NN model’s weights and biases, aiming to minimize the MSE^84^.

## Supplementary Information

Supplementary Video 1.

Supplementary Information 1.

Supplementary Legends.

## Data Availability

The data obtained and analyzed throughout the research can be readily accessed via the GitHub and Zenodo repositories using the provided link: https://github.com/ehsan20e20e/SquareRR_AOPS/releases. 10.5281/zenodo.10850611. The GitHub repository can be referenced for Supporting code associated with the study: https://github.com/ehsan20e20e/SquareRR_AOPS. All the code and data are provided and made publicly available under MIT license for public access.

## References

[1] Yu, N. & Capasso, F. Flat optics with designer metasurfaces. *Nat. Mater.* **13**, 139–150. 10.1038/nmat3839 (2014). 24452357 10.1038/nmat3839

[2] Jafari, B. *et al.* Highly sensitive label-free biosensor: graphene/CaF2 multilayer for gas, cancer, virus, and diabetes detection with enhanced quality factor and figure of merit. *Sci. Rep.* **13**, 16184. 10.1038/s41598-023-43480-5 (2023). 37758823 10.1038/s41598-023-43480-5 PMC10533514

[3] Kildishev, A. V., Boltasseva, A. & Shalaev, V. M. Planar photonics with metasurfaces. *Science* **339**, 1232009. 10.1126/science.1232009 (2013). 23493714 10.1126/science.1232009

[4] Phetsang, S. *et al.* Copper/reduced graphene oxide film modified electrode for non-enzymatic glucose sensing application. *Sci. Rep.* **11**, 9302. 10.1038/s41598-021-88747-x (2021). 33927300 10.1038/s41598-021-88747-x PMC8085015

[5] Zare, A.-A., Naderi-Manesh, H., Naghib, S. M., Shamsipur, M. & Molaabasi, F. Label-free electrochemical cancer cell detection leveraging hemoglobin-encapsulated silver nanoclusters and Cu-MOF nanohybrids on a graphene-assisted dual-modal probe. *Sci. Rep.* **13**, 21980. 10.1038/s41598-023-49418-1 (2023). 38082024 10.1038/s41598-023-49418-1 PMC10713537

[6] Kang, L. *et al.* Nonlinear chiral meta-mirrors: Enabling technology for ultrafast switching of light polarization. *Nano Lett.* **20**, 2047–2055. 10.1021/acs.nanolett.0c00007 (2020). 32031817 10.1021/acs.nanolett.0c00007

[7] Kang, L., Jenkins, R. P. & Werner, D. H. Recent progress in active optical metasurfaces. *Adv. Opt. Mater.* **7**, 1801813. 10.1002/adom.201801813 (2019). 10.1002/adom.201801813

[8] Kang, L., Zhao, Q., Zhao, H. & Zhou, J. Magnetically tunable negative permeability metamaterial composed by split ring resonators and ferrite rods. *Opt. Express* **16**, 8825–8834. 10.1364/OE.16.008825 (2008). 18545595 10.1364/OE.16.008825

[9] Betzig, E. *et al.* Imaging intracellular fluorescent proteins at nanometer resolution. *Science* **313**, 1642–1645. 10.1126/science.1127344 (2006). 16902090 10.1126/science.1127344

[10] Rust, M. J., Bates, M. & Zhuang, X. Sub-diffraction-limit imaging by stochastic optical reconstruction microscopy (STORM). *Nat. Methods* **3**, 793–796. 10.1038/nmeth929 (2006). 16896339 10.1038/nmeth929 PMC2700296

[11] Hess, S. T., Girirajan, T. P. & Mason, M. D. Ultra-high resolution imaging by fluorescence photoactivation localization microscopy. *Biophys. J.* **91**, 4258–4272. 10.1529/biophysj.106.091116 (2006). 16980368 10.1529/biophysj.106.091116 PMC1635685

[12] Hirbodvash, Z. et al. Infrared surface plasmons on a Au waveguide electrode open new redox channels associated with the transfer of energetic carriers. *Sci. Adv.* **8**, eabm9303. 10.1126/sciadv.abm9303 (2022). 10.1126/sciadv.abm9303 PMC9116605 35584214

[13] Jin, J.-M. *The finite element method in electromagnetics*. (John Wiley & Sons, 2015).

[14] Taflove, A., Hagness, S. C. & Piket-May, M. Computational electromagnetics: The finite-difference time-domain method. *Electr. Eng. Handb.* **3**, 15. 10.1016/b978-012170960-0/50046-3 (2005). 10.1016/b978-012170960-0/50046-3

[15] Zimmerman, W. B. *Multiphysics modeling with finite element methods*. Vol. 18 (World Scientific Publishing Company, 2006).

[16] Selmy, A. E., Soliman, M. & Allam, N. K. Refractory plasmonics boost the performance of thin-film solar cells. *Emerg. Mater.* **1**, 185–191. 10.1007/s42247-018-0017-x (2018). 10.1007/s42247-018-0017-x

[17] Turitsyn, S. K. *et al.* Nonlinear Fourier transform for optical data processing and transmission: Advances and perspectives. *Optica* **4**, 307–322. 10.1109/JLT.2021.3051609 (2017). 10.1109/JLT.2021.3051609

[18] LeCun, Y., Bengio, Y. & Hinton, G. Deep learning. *Nature* **521**, 436–444. 10.1038/nature14539 (2015). 26017442 10.1038/nature14539

[19] Fernández-Delgado, M. *et al.* An extensive experimental survey of regression methods. *Neural Netw.* **111**, 11–34. 10.1016/j.neunet.2018.12.010 (2019). 30654138 10.1016/j.neunet.2018.12.010

[20] Sakamoto, I., Okada, S., Nishiyama, N., Hu, X. & Amemiya, T. Deep learning improves performance of topological bending waveguides. *Opt. Express* **32**, 1286–1294. 10.1364/OE.507479 (2024). 38297683 10.1364/OE.507479

[21] Yeung, C., Pham, B., Zhang, Z., Fountaine, K. T. & Raman, A. P. Hybrid supervised and reinforcement learning for the design and optimization of nanophotonic structures. *Opt. Express* **32**, 9920–9930. 10.1364/OE.512159 (2024). 38571216 10.1364/OE.512159

[22] Farrokhi, M. *et al.* The AI diagnostician: Improving medical diagnosis with artificial intelligence. *Kindle* **4**, 1–219 (2024).

[23] Malheiros-Silveira, G. N. & Hernandez-Figueroa, H. E. Prediction of dispersion relation and PBGs in 2-D PCs by using artificial neural networks. *IEEE Photon. Technol. Lett.* **24**, 1799–1801. 10.1109/LPT.2012.2215846 (2012). 10.1109/LPT.2012.2215846

[24] El-Mosalmy, D. D., Hameed, M., Areed, N. F. & Obayya, S. Novel neural network based optimization approach for photonic devices. *Opt. Quant. Electron.* **46**, 439–453. 10.1007/s11082-013-9869-8 (2014). 10.1007/s11082-013-9869-8

[25] Andrawis, R. R., Swillam, M. A., El-Gamal, M. A. & Soliman, E. A. Artificial neural network modeling of plasmonic transmission lines. *Appl. Opt.* **55**, 2780–2790. 10.1364/AO.55.002780 (2016). 27139685 10.1364/AO.55.002780

[26] da Silva Ferreira, A., da Silva Santos, C. H., Gonçalves, M. S. & Figueroa, H. E. H. Towards an integrated evolutionary strategy and artificial neural network computational tool for designing photonic coupler devices. *Appl. Soft Comput.* **65**, 1–11. 10.1016/j.asoc.2017.12.043 (2018).

[27] da Silva Ferreira, A., Malheiros-Silveira, G. N. & Hernández-Figueroa, H. E. Computing optical properties of photonic crystals by using multilayer perceptron and extreme learning machine. *J. Lightw. Technol.* **36**, 4066–4073. 10.1109/JLT.2018.2856364 (2018).

[28] Asano, T. & Noda, S. Optimization of photonic crystal nanocavities based on deep learning. *Opt. Express* **26**, 32704–32717. 10.1364/OE.26.032704 (2018). 30645432 10.1364/OE.26.032704

[29] Gostimirovic, D. & Winnie, N. Y. An open-source artificial neural network model for polarization-insensitive silicon-on-insulator subwavelength grating couplers. *IEEE J. Select. Top. Quant. Electron.* **25**, 1–5. 10.1109/JSTQE.2018.2885486 (2018). 10.1109/JSTQE.2018.2885486

[30] Hammond, A. M. & Camacho, R. M. Designing integrated photonic devices using artificial neural networks. *Opt. Express* **27**, 29620–29638. 10.1364/OE.27.029620 (2019). 31684220 10.1364/OE.27.029620

[31] Chen, X. *et al.* Grating waveguides by machine learning for augmented reality. *Appl. Opt.* **62**, 2924–2935. 10.1364/AO.486285 (2023). 37133137 10.1364/AO.486285

[32] Peurifoy, J. *et al.* Nanophotonic particle simulation and inverse design using artificial neural networks. *Sci. Adv.* **4**, eaar4206. 10.1126/sciadv.aar4206 (2018). 10.1126/sciadv.aar4206 PMC5983917 29868640

[33] Sajedian, I., Kim, J. & Rho, J. Finding the optical properties of plasmonic structures by image processing using a combination of convolutional neural networks and recurrent neural networks. *Microsyst. Nanoeng.* **5**, 27. 10.1038/s41378-019-0069-y (2019). 31240107 10.1038/s41378-019-0069-y PMC6572799

[34] Adibnia, E., Mansouri-Birjandi, M. A., Ghadrdan, M. & Jafari, P. A deep learning method for empirical spectral prediction and inverse design of all-optical nonlinear plasmonic ring resonator switches. *Sci. Rep.* **14**, 5787. 10.1038/s41598-024-56522-3 (2024). 38461205 10.1038/s41598-024-56522-3 PMC10924975

[35] Liu, W. *et al.* A survey of deep neural network architectures and their applications. *Neurocomputing* **234**, 11–26. 10.1016/j.neucom.2016.12.038 (2017). 10.1016/j.neucom.2016.12.038

[36] Alzubaidi, L. *et al.* Review of deep learning: Concepts, CNN architectures, challenges, applications, future directions. *J. Big Data* **8**, 1–74. 10.1186/s40537-021-00444-8 (2021). 33816053 10.1186/s40537-021-00444-8 PMC8010506

[37] Chen, X.-W. & Lin, X. Big data deep learning: Challenges and perspectives. *IEEE Access* **2**, 514–525. 10.1109/ACCESS.2014.2325029 (2014). 10.1109/ACCESS.2014.2325029

[38] Johnson, J. M. & Khoshgoftaar, T. M. Survey on deep learning with class imbalance. *J. Big Data* **6**, 1–54. 10.1186/s40537-019-0192-5 (2019). 10.1186/s40537-019-0192-5

[39] Bejani, M. M. & Ghatee, M. A systematic review on overfitting control in shallow and deep neural networks. *Artif. Intell. Rev.* 1–48. 10.1007/s10462-021-09975-1 (2021).

[40] Rai, A. Explainable AI: From black box to glass box. *J. Acad. Mark. Sci.* **48**, 137–141. 10.1007/s11747-019-00710-5 (2020). 10.1007/s11747-019-00710-5

[41] von Eschenbach, W. J. Transparency and the black box problem: Why we do not trust AI. *Philos. Technol.* **34**, 1607–1622. 10.1007/s13347-021-00477-0 (2021). 10.1007/s13347-021-00477-0

[42] Chen, J. & Ran, X. Deep learning with edge computing: A review. *Proc. IEEE* **107**, 1655–1674. 10.1109/JPROC.2019.2921977 (2019). 10.1109/JPROC.2019.2921977

[43] Kljucaric, L. & George, A. D. Deep learning inferencing with high-performance hardware accelerators. *ACM Trans. Intell. Syst. Technol.* **14**, 1–25. 10.1145/3594221 (2023). 10.1145/3594221

[44] Chakraborty, A., Alam, M., Dey, V., Chattopadhyay, A. & Mukhopadhyay, D. A survey on adversarial attacks and defences. *CAAI Trans. Intell. Technol.* **6**, 25–45. 10.1049/cit2.12028 (2021). 10.1049/cit2.12028

[45] Theocharides, T., Shafique, M., Choi, J. & Mutlu, O. Guest editorial: Robust resource-constrained systems for machine learning. *IEEE Des. Test* **37**, 5–7. 10.1109/MDAT.2020.2971201 (2020). 10.1109/MDAT.2020.2971201

[46] Kang, L. *et al.* In *Metamaterials-by-Design* 167–201 (Elsevier, 2024). 10.1016/B978-0-32-399985-4.00014-3.

[47] Dory, C. *et al.* Inverse-designed diamond photonics. *Nat. Commun.* **10**, 3309. 10.1038/s41467-019-11343-1 (2019). 31346175 10.1038/s41467-019-11343-1 PMC6658519

[48] Liu, D., Tan, Y., Khoram, E. & Yu, Z. Training deep neural networks for the inverse design of nanophotonic structures. *Acs Photon.* **5**, 1365–1369. 10.1021/acsphotonics.7b01377 (2018). 10.1021/acsphotonics.7b01377

[49] Rakhshani, M. R. & Mansouri-Birjandi, M. A. High sensitivity plasmonic refractive index sensing and its application for human blood group identification. *Sensors Actuators B Chem.* **249**, 168–176. 10.1016/j.snb.2017.04.064 (2017). 10.1016/j.snb.2017.04.064

[50] Ghadrdan, M. & Mansouri-Birjandi, M. A. Design and implementation of optical switches based on nonlinear plasmonic ring resonators: circular, square and octagon. *Photon. Nanostruct. Fund. Appl.* **29**, 15–21. 10.1016/j.photonics.2018.01.003 (2018).

[51] Molesky, S. *et al.* Inverse design in nanophotonics. *Nat. Photon.* **12**, 659–670. 10.1038/s41566-018-0246-9 (2018). 10.1038/s41566-018-0246-9

[52] Kang, L., Wu, Y. & Werner, D. H. Nonlinear chiral metasurfaces based on the optical Kerr effect. *Adv. Opt. Mater.* **11**, 2202658. 10.1002/adom.202202658 (2023). 10.1002/adom.202202658

[53] Chung, S.-Y., Wang, C.-Y., Teng, C.-H., Chen, C.-P. & Chang, H.-C. Simulations of dielectric and plasmonic waveguide-coupled ring resonators using the legendre pseudospectral time-domain method. *J. Lightwave Technol.* **30**, 1733–1742. 10.1109/JLT.2012.2188851 (2012). 10.1109/JLT.2012.2188851

[54] Nozhat, N. & Granpayeh, N. All-optical nonlinear plasmonic ring resonator switches. *J. Mod. Opt.* **61**, 1690–1695. 10.1080/09500340.2014.951008 (2014). 10.1080/09500340.2014.951008

[55] Zand, I., Abrishamian, M. S. & Berini, P. Highly tunable nanoscale metal-insulator-metal split ring core ring resonators (SRCRRs). *Opt. Express* **21**, 79–86. 10.1364/OE.21.000079 (2013). 23388898 10.1364/OE.21.000079

[56] Pooretemad, S., Pav, M., Kashani, Z. G. & Granpayeh, N. Ultra-compact all-optical plasmonic switch for three telecommunication windows using a nonlinear Kerr material and Fano resonance. *Appl. Opt.* **62**, 4123–4133. 10.1364/AO.484012 (2023). 37706726 10.1364/AO.484012

[57] Cai, X., Xu, Q., Wang, S. & Li, S. Low-cross-talk and high-contrast all optical bistable switching based on coupled defects in a nonlinear photonic crystal cross-waveguide geometry. *Photon. Nanostruct. Fund. Appl.* **13**, 89–96. 10.1016/j.photonics.2014.11.001 (2015).

[58] Ghadrdan, M., Shahraki, M. & Mansouri-Birjandi, M. A. Plasmonic switch based on asymmetric cavities with embedding square of gold inside the cavities. *J. Nanophoton.* **17**, 036004–036004. 10.1117/1.JNP.17.036004 (2023). 10.1117/1.JNP.17.036004

[59] Moon, K. & Park, S. Graphene-based plasmonic switch using resonant coupling to the local plasmon resonance. *Phys. Rev. Appl.* **11**, 034074. 10.1103/PhysRevApplied.11.034074 (2019). 10.1103/PhysRevApplied.11.034074

[60] Zamani, M. Photonic crystal-based optical filters for operating in second and third optical fiber windows. *Superlattices Microstruct.* **92**, 157–165. 10.1016/j.spmi.2016.02.025 (2016). 10.1016/j.spmi.2016.02.025

[61] Hong, Y. *et al.* Numerical and experimental study on the impact of chromatic dispersion on O-band direct-detection transmission. *Appl. Opt.* **60**, 4383–4390. 10.1364/AO.424962 (2021). 34143128 10.1364/AO.424962

[62] Piggott, A. Y., Petykiewicz, J., Su, L. & Vučković, J. Fabrication-constrained nanophotonic inverse design. *Sci. Rep.* **7**, 1786. 10.1038/s41598-017-01939-2 (2017). 28496126 10.1038/s41598-017-01939-2 PMC5431915

[63] Verma, S., Chugh, S., Ghosh, S. & Rahman, B. A. A comprehensive deep learning method for empirical spectral prediction and its quantitative validation of nano-structured dimers. *Sci. Rep.* **13**, 1129. 10.1038/s41598-023-28076-3 (2023). 36670171 10.1038/s41598-023-28076-3 PMC9860028

[64] Singh, R., Agarwal, A. & Anthony, B. W. Design of optical meta-structures with applications to beam engineering using deep learning. *Sci. Rep.* **10**, 19923. 10.1038/s41598-020-76225-9 (2020). 33199746 10.1038/s41598-020-76225-9 PMC7669879

[65] Zhang, T. *et al.* Efficient spectrum prediction and inverse design for plasmonic waveguide systems based on artificial neural networks. *Photon. Res.* **7**, 368–380. 10.1364/PRJ.7.000368 (2019). 10.1364/PRJ.7.000368

[66] Singh, R., Agarwal, A. & Anthony, B. Mapping the design space of photonic topological states via deep learning. *Optics Express* **28**, 27893–27902. 10.1364/OE.398926 (2020). 10.1364/OE.398926 32988072

[67] Malkiel, I. et al. Plasmonic nanostructure design and characterization via deep learning. *Light: Sci. Appl.* **7**, 60. 10.1038/s41377-018-0060-7 (2018). 10.1038/s41377-018-0060-7 PMC6123479 30863544

[68] Li, X., Shu, J., Gu, W. & Gao, L. Deep neural network for plasmonic sensor modeling. *Optical Mater. Express* **9**, 3857–3862. 10.1364/OME.9.003857 (2019). 10.1364/OME.9.003857

[69] Wu, Q. *et al.* Deep neural network for designing near-and far-field properties in plasmonic antennas. *Opt. Mater. Express* **11**, 1907–1917. 10.1364/OME.428772 (2021). 10.1364/OME.428772

[70] Sandhibigraha, S., Mandal, S., Awasthi, M., Kanti Bandyopadhyay, T. & Bhunia, B. Optimization of various process parameters for biodegradation of 4-chlorophenol using Taguchi methodology. *Biocatal. Agric. Biotechnol.* **24**, 101568. 10.1016/j.bcab.2020.101568 (2020).

[71] Bilga, P. S., Singh, S. & Kumar, R. Optimization of energy consumption response parameters for turning operation using Taguchi method. *J. Clean. Prod.* **137**, 1406–1417. 10.1016/j.jclepro.2016.07.220 (2016). 10.1016/j.jclepro.2016.07.220

[72] Zaman, M. A. Photonic radiative cooler optimization using Taguchi’s method. *Int. J. Thermal Sci.* **144**, 21–26. 10.1016/j.ijthermalsci.2019.05.019 (2019). 10.1016/j.ijthermalsci.2019.05.019

[73] Ketkar, N. & Ketkar, N. Introduction to keras. In *Deep learning with python: A hands-on introduction*, 97–111. 10.1007/978-1-4842-2766-4_7 (2017).

[74] Singh, P., Manure, A., Singh, P. & Manure, A. Introduction to tensorflow 2.0. In *Learn TensorFlow 2.0: Implement Machine Learning and Deep Learning Models with Python*, 1–24. 10.1007/978-1-4842-5558-2_1 (2020).

[75] Van Rossum, G. & Drake, F. L. *Python reference manual*. Vol. 111 (Centrum voor Wiskunde en Informatica Amsterdam, 1995).

[76] Rolon-Mérette, D., Ross, M., Rolon-Mérette, T. & Church, K. Introduction to anaconda and python: Installation and setup. *Quant. Methods Psychol.* **16**, S3–S11. 10.20982/tqmp.16.5.S003 (2016).

[77] McKinney, W. Pandas, python data analysis library. *URL *http://pandas.pydata.org, 3–15 (2015).

[78] Pedregosa, F. *et al.* Scikit-learn: Machine learning in python. *J. Mach. Learn. Res.* **12**, 2825–2830 (2011).

[79] McKinney, W. *Python for data analysis: Data wrangling with Pandas, NumPy, and IPython* (" O'Reilly Media, Inc.", 2012).

[80] Maas, A. L., Hannun, A. Y. & Ng, A. Y. *Rectifier nonlinearities improve neural network acoustic models*. In *Proc. icml.* 3 (Atlanta, GA).

[81] Zhao, Y. et al. DeepAIR: A deep learning framework for effective integration of sequence and 3D structure to enable adaptive immune receptor analysis. *Sci. Adv. 9*, eabo5128. 10.1126/sciadv.abo5128 (2023). 10.1126/sciadv.abo5128 PMC10411891 37556545

[82] Chugh, S., Ghosh, S., Gulistan, A. & Rahman, B. Machine learning regression approach to the nanophotonic waveguide analyses. *J. Lightw. Technol.* **37**, 6080–6089. 10.1109/JLT.2019.2946572 (2019). 10.1109/JLT.2019.2946572

[83] Kinga, D. & Adam, J. B. *A method for stochastic optimization*. in *International conference on learning representations (ICLR).* 6 (San Diego, California;).

[84] Wilson, A. C., Roelofs, R., Stern, M., Srebro, N. & Recht, B. The marginal value of adaptive gradient methods in machine learning. *Adv. Neural Inf. Process. Syst.* **30**, 1 (2017).

