# Supplementary material for: Nanophotonic structure inverse design for switching application using deep learning
Source: Sci Rep. 2024 Sep 10;14:21094. doi: 10.1038/s41598-024-72125-4 (PMC11387741; doi:10.1038/s41598-024-72125-4)
Supplement: Supplementary file 2 — Supplementary Information 1. [file 41598_2024_72125_MOESM2_ESM.pdf]

scientific reports

Supplementary Information for

Nanophotonic Structure Inverse Design for Switching Application Using Deep Learning

Ehsan Adibnia *et al*.

Corresponding author: Mohammad Ali Mansouri-Birjandi
E-mail: mansouri@ece.usb.ac.ir

This PDF file includes:

Sections S1 to S7
Figs. S1 to S9
Tables S1 to S3
References (1 to 12)
DOI: 10.5281/zenodo.13191254.

SUPPLEMENTARY TEXT

Section S1: Taguchi method

In the present investigation, the Taguchi method was employed for the experimental design, leveraging its statistical capabilities to delineate the influence of various process parameters and precisely delineate the relationship between input and output variables. This method is superior to traditional approaches, such as the Factorial design, by obviating the need for extensive experiments, efficiently conserving time, and pinpointing optimal conditions.

We conducted the analysis using three distinct criteria: "lower is better," "higher is better," and "nominal is better" in order to evaluate a property of interest. Given the study's objectives, which included achieving a high contrast between the ports and a nominal resonance wavelength of 1550 nm, the statistical analyses adopted the "higher is better" criterion for contrast and the "nominal is better" criterion for resonance wavelength. We quantified the influence of each parameter on the contrast ratio and resonance wavelength by computing average values. Subsequently, we performed an Analysis of Variance (ANOVA) to determine the significance and contribution of each parameter to the output characteristics, precisely the contrast between the ports and the nominal resonance wavelength. The optimal experimental conditions were predicted, drawing on the insights from average values and ANOVA results.

This research scrutinized the effects of five parameters, each varied across four levels, encompassing the widths of the bus, drop, and square waveguides (30, 40, 50, and 60 nm), and the gaps within the bus and drop waveguides (15, 18, 21, and 25 nm). In contrast with the factorial design, which necessitates 1024 experiments, the Taguchi method significantly reduces the workload to just 16 runs, as detailed in Table S1, exemplifying its efficiency and efficacy in experimental design. The study's contrast ratio between the ports and the resonance wavelength was meticulously measured across sixteen simulations, adhering to the conditions delineated in Table S1.

This measurement aimed to elucidate the influence of structural parameters on the resonance characteristics of the system. The main effects plots, illustrated in Figure S1, reveal the variation in the average values of the contrast ratio and resonance wavelength about the structural parameters, offering insights into identifying optimal processing conditions. Furthermore, the ANOVA outcomes for the contrast ratio and resonance wavelength of the All-Optical Plasmonic Switch (AOPS) are detailed in Tables S2 and S3, respectively. These results highlight the relative significance of the width of the bus (W_{bus}), drop (W_{drop}), and square (W_{square}) waveguides, as well as the gaps within the bus (G_{bus}) and drop (G_{drop}) waveguides on the contrast ratio, estimated at approximately 4%, 19%, 23%, 47%, and 8% respectively. In a parallel vein, their significance on the resonance wavelength is quantified at approximately 51%, 16%, 19%, 12%, and 3%, respectively. Intriguingly, the inner pie chart in Figure S2A visually represents the significance of each structural parameter on the contrast ratio, whereas the outer pie chart delineates their impact on the resonance wavelength. As illustrated in Figure S2B, the pie chart further distills that the average significance attributed to these parameters is approximately 21%, 17%, 21%, 29%, and 5%.

Given these analytical outcomes and considering the computational expenditure associated with varying structural parameters, a definitive conclusion emerges. Specifically, the gaps within the bus and drop waveguides exert the most profound impact on the resonance characteristics of AOPS and the efficiency of Neural Network (NN) training, underscoring their pivotal role in optimizing the system's performance. The findings resulting from the application of the Taguchi method, along with the incorporation of computational cost considerations, have been visually represented in Figure S3.

In the concluding segment of this investigation, the primary aim—leveraging the Taguchi robust design methodology to substantially diminish the variance surrounding the target metrics of product performance characteristics—was successfully realized. Determining optimal values for significant parameters, which effectively mitigated the impact of insignificant factors, facilitated the enhancement of the NN's quality attributes. Implementing Taguchi's robust design principles reduced the temporal and financial resources required for experimental procedures.

By selecting an appropriate orthogonal array, the study necessitated merely 16 experiments within the context of resonance characteristics, as opposed to the 1024 that a comprehensive factorial design approach would entail. This strategic selection yielded optimal outcomes, rendering the additional 1008 experiments superfluous. Consequently, this approach not only conserved time and costs but also elevated the quality of the output.

Section S2: Dataset discussion

This section summarizes the training dataset used to train the deep NN. The training dataset is crucial for revealing concealed trends and facilitating precise predictions. Using a complete and accurate training dataset, the deep NN displays its proficiency in dealing with differing inputs and allocating correct weightings via each neuron's activation function. Figure S4 provides two complementary views of our single training dataset. As can be seen, Figure S4A shows the input training dataset used to train the established NN. The y-axis in both figures represents the values of these variables after standardization or normalization. It's important to note that this is a single, unified dataset used to train our deep NN model for predicting both drop port and through port spectra simultaneously. This approach allows our model to capture the full spectral behavior of the device in a single training process, enhancing computational efficiency and ensuring consistency in predictions across both ports. Our training set comprises 18,432 unique structures (number of simulations), each with 800 corresponding wavelength values that lead to 14.7 million individual data points (18,432 × 800 = 14,745,600).

The dataset includes various parameters: S_1 denotes the width of the bus waveguide, S_2 indicates the width of the drop waveguide, S_3 stands for the square waveguide width, S_4 is the gap in the bus waveguide, and S_5 is the gap in the drop waveguide. These parameters are showcased using distinct blue, orange, green, red, and violet bars in Figure S4A. From a different perspective, Figure S4B demonstrates the output dataset, which aids in the training and predicting outcomes with the constructed deep NN.

This dataset includes spectral data from the Finite-Difference Time-Domain (FDTD) methodology, illustrating the transmission spectra for both the through and drop ports. In

Figure S4B, the blue bars illustrate the through-port transmission spectra, and the orange bars show the drop-port transmission spectra. The output dataset is used as an objective when training and assessing the effectiveness of the deep NN. According to the depiction in Figure S4, the information represents a remarkably fair and steady spread.

In the histogram, data points are evenly distributed within different bins, suggesting that the input and output datasets are extensively spread out, lacking significant bias or unevenness. The emphasis on the diversity and representativeness of datasets through an equitable distribution offers a noteworthy opportunity to develop and enhance the NN model's generalization capabilities.

Section S3: Optimizing hyperparameters of deep NN

This section is dedicated to fine-tuning hyperparameters within a deep NN to enhance its predictive accuracy as measured by the Mean Squared Error (MSE). A consistent learning rate of 0.1 was maintained across all predictive models to ensure peak efficacy. Displayed in Figure S5A is a graph depicting the MSE outcomes for an NN configured with 160 neurons in the central layer and trained over 1000 epochs, with the independent variable being the count of hidden layers. The results indicate that a traditional NN with a single hidden layer is inadequate for proficient prediction tasks, as it lacks the requisite complexity to model the nuanced correlations within the dataset.

This insufficiency underscores the shortfalls of less complex neural architectures when confronted with sophisticated, nonlinear challenges. Conversely, NNs with greater depth, like the one applied in this research, demonstrate superior capability in identifying and assimilating complex features, which is essential for complex predictive tasks. An increase in epochs correlates with a decline in MSE values. Furthermore, there is a notable decrease in MSE as the hidden layer count ascends, stabilizing at the juncture of 13 layers. Consequently, an NN structure with 11 hidden layers was selected for optimal hyperparameter configuration, striking a balance between low MSE outcomes and reduced computational demands.

This study aimed to enhance the operational effectiveness of the deep NN by meticulously selecting optimal hyperparameters. Initially, the research methodology entailed establishing a correlation between the neuron count (N_n) in the central layer and the total number of dependent hidden layers (N_l), utilizing the formula $N_n = 2^{(N_l-1)/2}$. This investigation extended to examining neuron distributions across various scales, guided by the Fibonacci sequence. The impact of augmenting neuron quantities and increasing the number of epochs on the MSE metrics across 9, 11, and 13 hidden layers was meticulously documented, as illustrated in Figures S5B, C, and D. To refine deep NN performance, a series of neuron configurations following the Fibonacci sequence—comprising one, two, three, five, and eight neurons—underwent rigorous testing. The corresponding MSE outcomes for these configurations were delineated through violet, yellow, orange, blue, and green trajectories, respectively.

Upon thorough evaluation, a configuration employing one hundred and sixty neurons in the central hidden layer was selected, predicated on its demonstrably superior performance characterized by diminished MSE values and enhanced convergence rates. This strategic

choice proved pivotal in refining the deep NN's operational efficiency and pinpointing the hyperparameters that yield the most favorable outcomes. To ensure a comprehensive assessment of the deep NN's capabilities, we steadfastly maintained the epoch count at 1000, with the neuron count in the central layer fixed at 160. This approach facilitated an exhaustive exploration of the deep NN's functionality, underscoring the critical role of hyperparameter optimization in advancing deep learning (DL) technologies. In the initial stages of our investigation, we developed predictions based on specific design input parameters. These parameters saw S_1, S_2, and S_3 settings at 45 nm and S_4 and S_5 at 20 nm. Following this, we anticipated the transmission spectra at the through and drop ports. Figure S6 comprehensively compares the actual spectra against the NN's predicted values for evaluating both ports.

Within each segment of Figure S6, the integrity of the transmission spectra derived from the FDTD method is represented by a continuous orange line. Meanwhile, the predictions made by the deep NN are marked by a dashed blue line. The analysis of the through port spectrum is illustrated on the left side of Figure S6, whereas the analysis of the drop port spectrum is shown on the right. The subfigures, designated as S6A/B, S6C/D, S6E/F, S6G/H, S6I/J, S6K/L, and S6M/N, display the transmission spectra for an ascending series of hidden layers within the network, specifically 1, 3, 5, 7, 9, 11, and 13 hidden layers, in the specified sequence. These illustrations visually compare the experimentally obtained spectra and those predicted by the deep NN for each configuration. The meticulous adjustment of hyperparameters, such as the number of hidden layers and the number of neurons, significantly enhances the analytical accuracy of the deep NN. This iterative refinement process enables the model to comprehend and articulate the intricate relationships present within the dataset. The insights gleaned from the predictions produced by this enhanced model are invaluable for understanding the operational dynamics of the system under study. This account underscores the critical importance of hyperparameter optimization in deep NNs. It demonstrates the effectiveness of the chosen parameters in the precise prediction of transmission spectra, engaging the reader in the intricacies of computational modeling and its implications for the field of nanophotonics.

Section S4: The strategy for finding a sharper dip in the transmission spectrum

This segment details the evaluation of a novel strategy designed to highlight a transmission spectrum with a distinctly deeper trough. The investigative lens is centered on the second-order derivative of the transmission spectrum, quantitatively expressed as $\mathrm{d}^2x/\mathrm{d}\lambda^2$. Figure S7 showcases that an increase in the peak of the second derivative graph (Figure S7A) indicates a more acute dip within the transmission spectrum (Figure S7B). Such a strong association between the second derivative graph and the transmission spectrum provides an enhanced understanding of spectral properties, pivotal for conceptualizing and enhancing nanophotonic constructs.

This section graphed the transmission spectra for a series of four successive structures. The transmission spectra depicted in Figure S7 were captured utilizing the FDTD approach to reduce the potential discrepancies that DL algorithms might introduce.

Section S5: Design and Simulation of AOPS

S5.1: Electromagnetic theory of AOPS design and modeling

This research builds upon the foundational successes of previous endeavors in configuring and operationalizing AOPSs, integrating a design that incorporates materials and configurations known for their superior performance in switching applications. Advances in manufacturing technologies now permit the realization of this design[1,2], which features a circular resonator interconnected with input and output waveguides of varying widths. The depicted AOPS structures, as illustrated in Figure 2 (in the main manuscript), comprise a silver film layered with a low refractive index material, specifically an Au/SiO_2 composite. This composite, renowned for its pronounced Kerr nonlinearity, employs an Au/SiO_2 blend where the refractive index (n_0) is 1.47, and the Kerr nonlinear coefficient (n_2) is 2.07×10^{-9} cm^2/W[3], facilitating the generation of surface plasmon resonance phenomena that significantly enhance third-order optical nonlinear susceptibility. The AOPS's cladding layer is air, and its foundational layer is made of glass. In pursuit of practical fabrication, the silver and Au/SiO_2 layers each maintain a thickness of 50 nm, complemented by a 100 nm thick glass film serving as the substrate. The resonant wavelengths (λ_r) are calculated through the following equation:

$$\lambda_{\mathrm{r}} = L n_{\mathrm{eff}}/m \tag{S1}$$

Where L denotes the effective length, n_{eff} the effective refractive index, and m is an integer. The Kerr effect accounts for the observed nonlinearity in ring resonators, with the refractive index adjusting according to both the Kerr nonlinear coefficient and the intensity of the incident light, as expounded in the reference[4].

$$n = n_0 + n_2 I \tag{S2}$$

In this equation, n_0 symbolizes the linear refractive index, n_2 stands for the nonlinear refractive index coefficient, and I signifies the intensity of light.

Surface plasmon polaritons, excited primarily through transverse magnetic polarization in metallic structures[5], exhibit remarkable electromagnetic field intensification, confinement beyond the wavelength scale, and heightened sensitivity to adjacent dielectric environments. These properties render surface plasmon polaritons particularly valuable for biosensing[6,7], nanophotonics[8,9], and metamaterials[10] applications. The engagement with TM-polarized light at resonant wavelengths triggers the excitation of surface plasmon polaritons within the ring resonator structure, with silver selected for its advantageous complex dielectric constant, as delineated by the Drude model[1,11].

$$\varepsilon(\omega) = \varepsilon_\infty - \omega_{\mathrm{p}}^2/\left(\omega^2 - j\gamma_{\mathrm{p}}\omega\right) \tag{S3}$$

In this context, ε_∞ equals 1.95, the relative permittivity occurring at infinite frequency. The angular frequency of the incident Lightwave is represented by ω. Also, γ_p, defined as the electron collision frequency, is quantified as 2×10^{13} rad/s while the plasma frequency, ω_p, is quantified as 1.37×10^{16} rad/s.

The division of the dielectric constant into real and imaginary parts allows the Drude-Lorentz model to be applied across various domains. Notably, it characterizes electronic transitions in metals and elucidates surface plasmon-polariton resonance phenomena. The

Drude-Lorentz model, which adequately illustrates the actions of free electrons in metals and their impact on surface plasmon resonance, is instrumental in identifying the electronic transition in metals. It is especially well-suited for substances that contain a greater ratio of free electrons than bound electrons. The computation of the dielectric constant, which can be separated into its real and imaginary parts, uses Drude's free-electron model. The dielectric constant can be explicated as:

$$\varepsilon_{\text{real}} = 1 - \frac{\omega_p^2 \tau^2}{1 + \omega^2 \tau^2} \tag{S4}$$

$$\varepsilon_{\text{imag}} = \frac{\omega_p^2 \tau}{\omega(\omega + \omega^2 \tau^2)} \tag{S5}$$

Under the context of metals in the vicinity of the near-infrared frequency range where the angular frequency (ω) significantly surpasses the inverse of the relaxation time ($1/\tau$), we resort to the methodology suggested by Johnson and Christy for the simplification of Eq. (S1) and Eq. (S2). The dielectric constant can be simplified and represented by:

$$\varepsilon(\omega) = 1 + \frac{\omega_p^2}{\omega^2} + j\frac{\omega_p^2}{\omega^3 \tau} = \varepsilon_{\text{real}} + \varepsilon_{\text{imag}} \tag{S6}$$

In such a specific case, the complex relative permittivity ($\varepsilon(\omega)$) is defined by its real portion ($\varepsilon_{\text{real}}$) and its imaginary portion ($\varepsilon_{\text{imag}}$). On the other hand, the complex refractive index (n) is composed of its real (n) and imaginary (κ) parts. The following relationship exists between these quantities[12]:

$$\varepsilon_{\text{real}} = n^2 - \kappa^2 \tag{S7}$$

$$\varepsilon_{\text{imag}} = 2n\kappa \tag{S8}$$

The Johnson and Christy methodology allowed us to determine the actual and imaginary parts of the complex refractive index. These components are then employed to compute the coefficients of transmission and reflection.

To explore the intrinsic physical properties of plasmonics, we use the FDTD method as a computational approach for solving the classic Maxwell's equations. The aforementioned analytical method aptly considers the electric field's sinusoidal time dependency. This time dependency is expressed as $E(r,t) = E(r)e^{-j\omega t}$. The process of deriving the Helmholtz equation from Maxwell's basic equations results in the appearance of the following mathematical expression:

$$\nabla^2 E + k_0^2 \varepsilon E = 0 \tag{S9}$$

The wavevector is symbolized as k_0, with the propagating field being elucidated by the formula $E(x,y,z) = E(x,y)e^{j\beta z}$. β corresponds to the propagation constant in this context.

The propagation constant is written in complex form as $\gamma = \alpha + j\beta$, where α denotes the attenuation constant. If α is zero, the problematic term, $\gamma = j\beta$, describes the exclusive dependency of propagation along the z-axis.

S5.2: FDTD simulation of AOPS

This study uses the FDTD numerical method to explore the proposed configurations' linear and nonlinear optical properties, facilitating an accurate simulation of the system's visual response. To ensure enhanced spatial resolution in our simulations, we have meticulously chosen the mesh dimensions to be Δx = Δz = 0.5 nm. We have employed a perfectly matched layer as an absorptive boundary condition to mitigate the undesirable effects of reflections at the simulation domain's boundaries. The perfectly matched layer showcases remarkable efficacy in absorbing outgoing waves and significantly reducing reflections, thereby augmenting the accuracy of our results. The architecture of the AOPS incorporated a silver film, which was coupled with an Au/SiO_2 layer possessing a low refractive index. This strategy facilitated control over SPPs and thereby enabled the desired switching functionality. The cladding layer, composed of air, and the base, made of glass, were selected for their beneficial optical properties, making them common choices in plasmonic device fabrication. Determining a 50 nm thickness for both the silver film and Au/SiO_2 layer stemmed from optimizing device efficiency, considering the impact of manufacturing processes on device performance.

Furthermore, integrating a 100 nm glass substrate augmented the device's mechanical support and stability. Applying the FDTD method allowed for a thorough investigation into the complex AOPS configuration's linear and nonlinear dynamics, allowing us to conduct a detailed analysis of the system's optical behavior. This ability, in turn, provided us with critical insights and a comprehensive understanding of its unique attributes and behavior under various conditions.

Section S6: Deep NN formulation

With the AOPS structural parameters elucidated, attention shifts toward developing mathematical models for forward and inverse analyses. These models are instrumental in defining the NN's problem space and establishing a correlation between input variables and resultant transmission spectra. A forward model is constructed by assigning specific structural design parameters (S_i) for the AOPS to predict the transmission spectrum across both through and drop ports. Employing a mapping function ($\mathbf{M}$) alongside input variables enables the derivation of transmission spectra (T_1 and T_2) for the respective ports:

$$\mathrm{T}_j^{\lambda} = \mathbf{M}\left(S_1 = \mathrm{W_{bus}}, \mathrm{S}_2 = \mathrm{W_{drop}}, S_3 = \mathrm{W_{square}}, S_4 = \mathrm{G_{bus}}, S_5 = \mathrm{G_{drop}}, S_6 = \lambda\right) \qquad \text{(S10)}$$

In this context, $\mathrm{W_{bus}}$ stands for the bus waveguide width, $\mathrm{W_{drop}}$ corresponds to the drop waveguide width, $\mathrm{W_{square}}$ designates the square ring waveguide width, $\mathrm{G_{bus}}$ is the bus waveguide gap, $\mathrm{G_{drop}}$ represents the drop waveguide gap, and λ is the input light wavelength, varying from λ = 1000 nm to 1800 nm.

In the established mathematical framework, we have incorporated the wavelength of the input light into the NN's forward model. This consideration has allowed it to predict transmittance values at both discrete and broadband wavelengths.

$$\min_{i=1,2,\dots,6}\left\{\left\|\widehat{\mathbf{M}}(S_i) - \mathrm{g}\right\|\right\} \qquad \text{(S11)}$$

In the equation above, $\widehat{\mathbf{M}}$ is a symbol signifying the predicted function, and the notation g stands for the ground truth, in other words, the factual observed values. It can, therefore, be

inferred that the problem in DL lies in discovering the perfect estimator for $\widehat{\mathbf{M}}$ using the existing training data.

Conversely, an inverse problem seeks to rebuild the design space using the provided transmission values. Rather than employing traditional approaches, our plan unveils a distinctive strategy. We establish a design space based on the transmission spectra represented by Eq. (S9), in which T_j denotes the transmission spectra. This work can be accomplished using the inverse function below:

$$S_i = \mathbf{M}^{-1}(T_j) \tag{S12}$$

The distinguishing aspect of our research is the awareness of a widespread challenge in the field, where traditional analytical techniques often prove insufficient in deriving the inverse function for AOPS configurations. In these scenarios, we present an innovative perspective by championing the employment of DL strategies as an alternative course of action. DL offers an influential mechanism for nonlinear approximation, with our study trailblazing an enhanced method to tackle both forward and inverse models. This cutting-edge approach fosters a thorough comprehension of the complex behavior of AOPS, signifying a remarkable advancement in the field.

Section S7: Analysis of the computational cost

S7.1: Comparison of the computational cost related to NNs with various architectures.

In this analysis, the focus shifts towards evaluating the efficiency of the devised NN in the context of its computational expenditure. Generating expansive training datasets for deep NNs necessitates substantial computational resources, introducing challenges in autonomously augmenting data points, especially for underrepresented areas absent from current datasets. This situation becomes more pronounced when considering the constraints on computational resources and the extent of researcher involvement in data curation, magnifying the hurdles faced.

Exploring methods for effectively enhancing datasets while preserving model generalization is crucial for ongoing research. Figure S8 aids in understanding the variances in training execution times, which are contingent on the number of neurons and hidden layers. This assessment calculates the computational costs of processing a single transmission spectrum during deep NN training, adjusting the neuron count across various hidden layer configurations. The results reveal that an escalation in the complexity of deep NN architectures and neuron numbers leads to an increased demand for computational resources.

It is noteworthy, however, that a deep NN configuration comprising eleven layers with 160 neurons in the central layer exhibits no further reduction in the loss function upon adding more layers and neurons, potentially precipitating the vanishing gradient dilemma. This observation implies that the deep NN demands additional computational resources and time to refine its predictions as the training progresses. Remarkably, the performance of the deep NN surpasses that of traditional FDTD solvers, which require thousands of seconds to compute plasmonic ring resonator optical responses using automated meshes. Although the initial creation of a comprehensive dataset via computationally intensive electromagnetic

simulations is unavoidable, this preprocessing is a one-time requirement. Furthermore, the application of the Taguchi method, as detailed in Section S1, presents an avenue for significantly reducing the volume of data needed for network training.

Post-training, the deep NN offers rapid predictions for novel configurations, indicating the potential to accelerate computational tasks upon the completion of training. These findings underscore a nuanced equilibrium between computational cost and predictive accuracy within deep NN architecture. In selecting the most suitable configuration for a specific task, it is imperative to judiciously evaluate the available computational resources against the desired precision levels. The quest for an optimal balance between computational expenditure and prediction accuracy remains a pivotal subject for further exploration, highlighting the interplay between efficiency and efficacy in deep NN development.

S7.2: Comparison of the computational cost between DL and numerical method.

In this investigation, we aim to scrutinize the computational costs associated with DL techniques in comparison to numerical methods such as the Finite Element Method (FEM) and the FDTD method. Figure S9 elucidates the computational expenses, quantified in seconds, incurred by the FEM and FDTD methods across a spectrum of meshing strategies. The essence of this analysis is to assess the computational burden of the DL paradigm vis-à-vis FEM and FDTD techniques. Specifically, Figure S9 illustrates the CPU time, in seconds, requisite for executing the FEM and FDTD methodologies under varied mesh configurations. Employing the most refined mesh size necessitated computational durations of 5871 seconds for FEM and 4515 seconds for FDTD, respectively.

For the FDTD method, the computational time was increased progressively to 4, 10, 18, 49, 124, 309, 941, and 2347 seconds for mesh levels classified from extremely coarse to extra fine, respectively. Similarly, for the FEM approach, computational times escalated to 5, 12, 23, 64, 161, 402, 1223, and 3051 seconds across the same mesh gradations. In stark contrast, implementing the DL methodology reduced the computational time to a mere fraction of a second, heralding a significant reduction in time consumption and offering substantial efficiency gains for practitioners.

Our research introduces an innovative strategy whereby a deep NN is adeptly trained to approximate spectral data, thereby markedly diminishing the processing time associated with the DL technique. By amalgamating the temporal metrics of these individual components, the cumulative time required for task completion can be precisely determined. Consequently, this approach affords the capability to ascertain execution times for batches or extended epochs, thereby enabling the identification of optimal techniques and models for various complex photonic devices.

This comparative analysis accentuates the formidable computational efficiency of the DL method, especially when juxtaposed with the FEM and FDTD methodologies across an array of mesh densities. The substantial diminution in processing time engendered by the DL approach paves the way for enhanced and more expeditious analysis of photonic devices, thereby fostering heightened productivity and innovation within the field.

FIGURES

Figure S1:

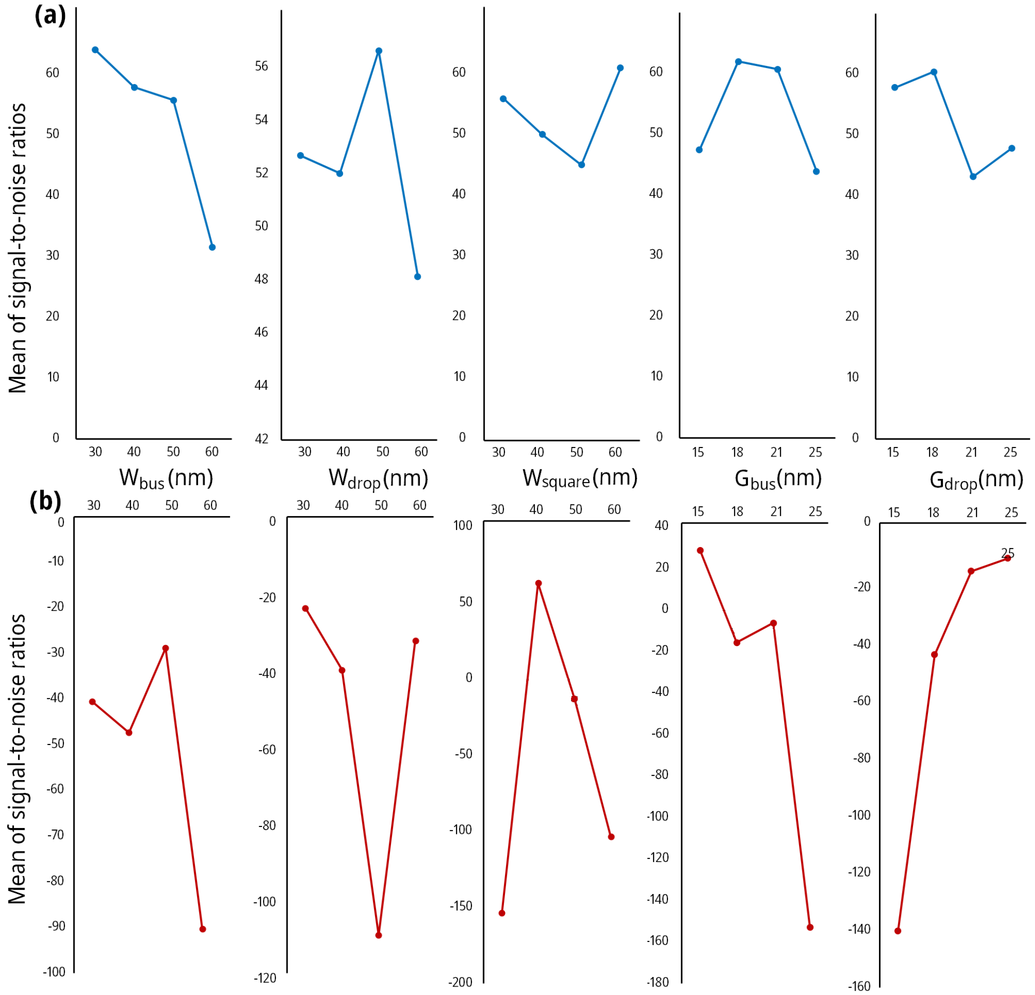

Figure S1. The main effect plots. The main effect analyses for the average value of five structural parameters on (**A**) the contrast ratio and (**B**) the resonance wavelength. The main effect analysis examines the individual influence of each parameter on the response variable while keeping other factors constant. It helps to identify the relative importance of each parameter in determining the quality or performance of the AOPS.

Figure S2:

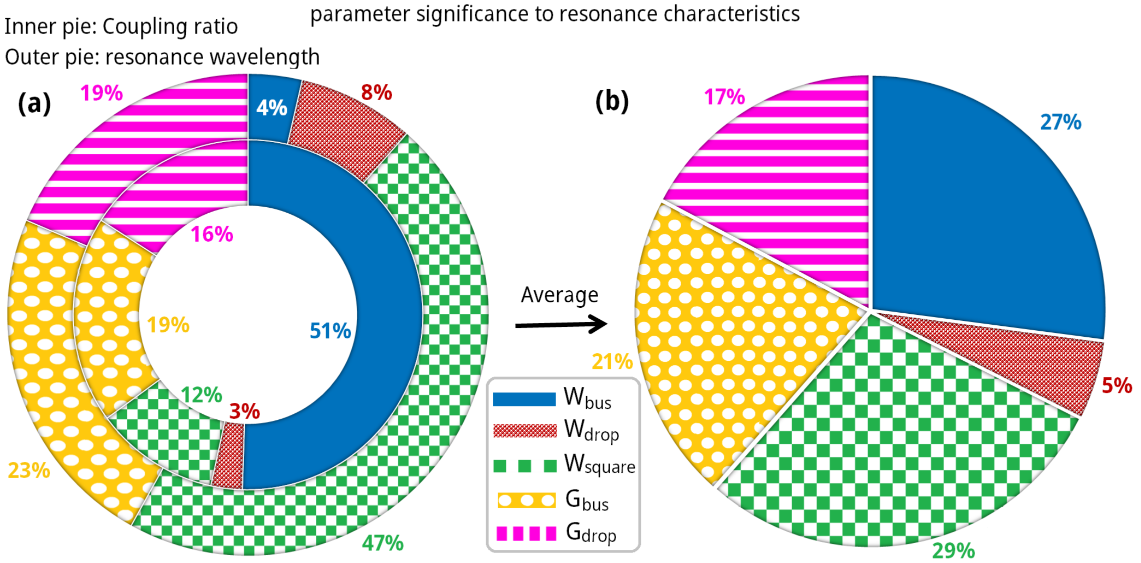

Figure S2. Pie charts depicting the significance of each structural parameter. In (**A**), the inner pie chart represents the significance of the contrast ratio, while the outer pie chart represents the significance associated with the resonance wavelength. Additionally, (**B**) displays the average of the two significance values.

Figure S3:

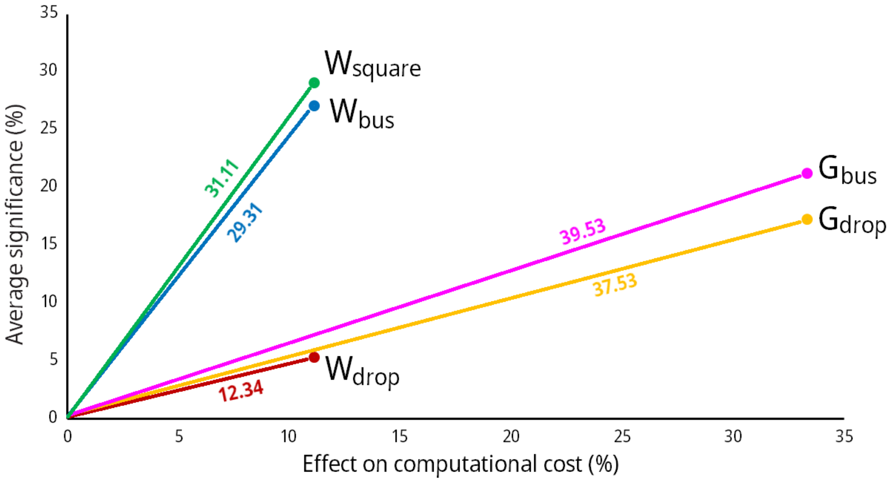

Figure S3. Visualizing the significance and computational cost. Visualization of the significance and computational cost considerations of each structural parameter related to resonance characteristics. S_4 and S_5 significantly influence the resonance properties of AOPS as well as the efficacy of NN training.

Figure S4:

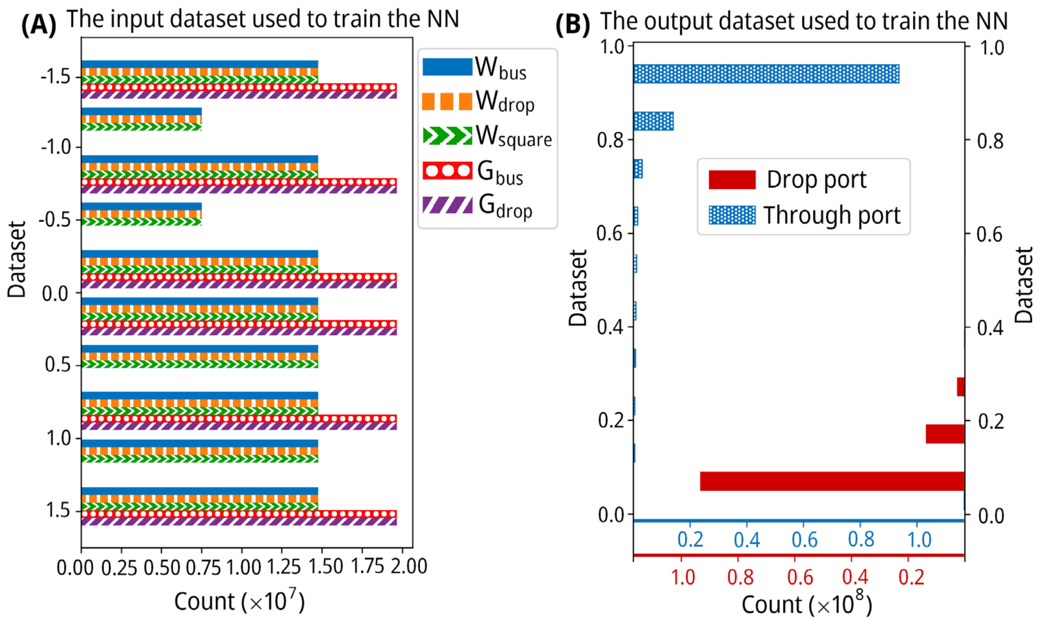

Figure S4. Histograms of the data distribution. Histograms convey the distribution of input and output variables for the square ring resonator model. (**A**) The standardized input data set employed in the NN training process is illustrated. This particular dataset encompasses various structural parameters of the resonator structure, including S_1, which denotes the width of the bus waveguide; S_2, the width of the drop waveguide; S_3, the width of the square waveguide; S_4, the gap of the bus waveguide; and S_5, the gap of the drop waveguide. The histogram elucidates the dispersion and occurrence rates of these input factors. (**B**) The progression of the output data set, applied in the training of the NN, is displayed. This data reflects the transmission spectra of the through and drop ports of the resonator. The histogram offers an illustrative summary of the transmission spectra's distribution and salient features for each port.

Figure S5:

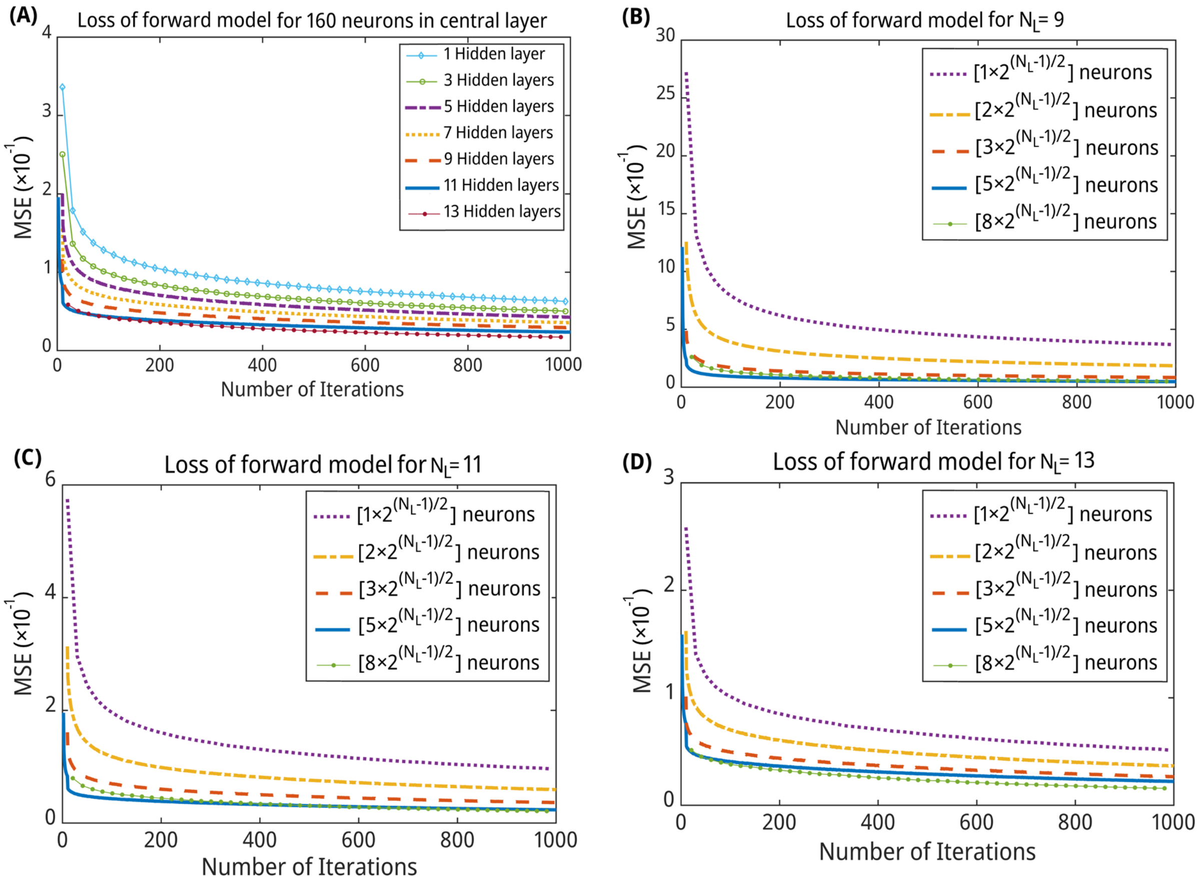

Figure S5. The MSE of validation across diverse configurations. The validation set loss metrics assessment across diverse configurations of hidden layers and neurons unfolds: (**A**) MSE metrics for various hidden layer configurations are meticulously documented, with a consistent neuron count of 160 in the central layer. This evaluation encompasses systems with hidden layers of 1, 2, 3, 4, 5, 6, and 12. Networks with limited layers exhibit constraints in capturing complex relationships, unlike their counterparts endowed with more layers, which leverage their enhanced hierarchical learning frameworks to identify more subtle features embedded within the dataset. Furthermore, the MSE metrics for different neuron count coefficients in the central layer, specifically at counts of 1, 2, 3, 5, and 8, are systematically recorded when the hidden layer count is held constant at (**B**) 9, (**C**) 11, and (**D**) 13. Observations indicate that networks with a single neuron may not adequately process intricate tasks. In contrast, increasing the neuron count amplifies a deep NN's analytical prowess. This enhancement facilitates the network's ability to detect finer patterns and correlations within the data, underscoring the pivotal role of neuron density in the central layer for optimizing network performance. This exploration not only elucidates the impact of architectural choices on the proficiency of deep NNs but also highlights the intricate balance between depth and neuron distribution as crucial determinants of model efficacy.

Figure S6:

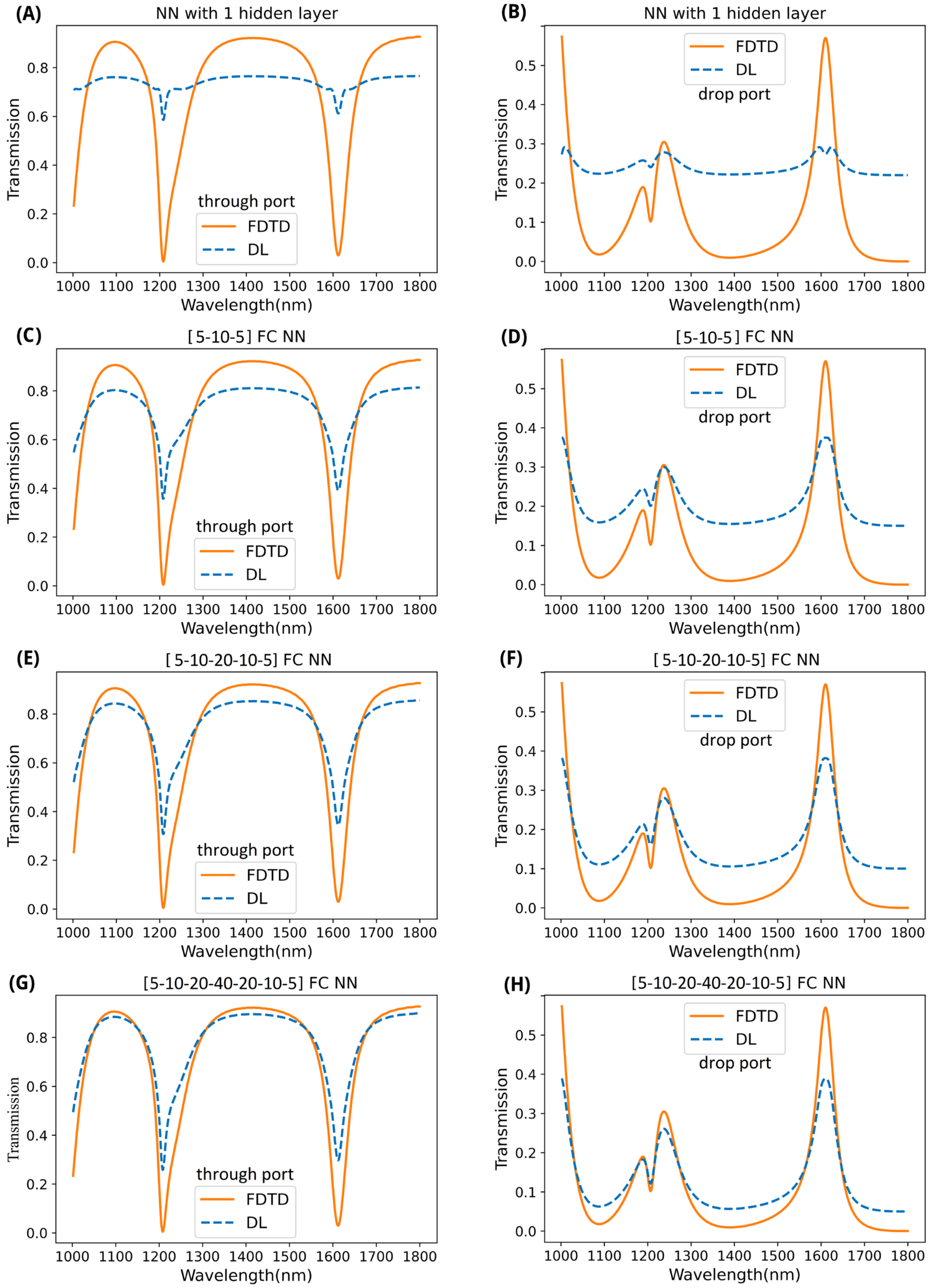
(A)
NN with 1 hidden layer
Transmission
Wavelength(nm)
through port
FDTD
DL
(B)
NN with 1 hidden layer
FDTD
DL
drop port
Transmission
Wavelength(nm)
(C)
[5-10-5] FC NN
Transmission
Wavelength(nm)
through port
FDTD
DL
(D)
[5-10-5] FC NN
FDTD
DL
drop port
Transmission
Wavelength(nm)
(E)
[5-10-20-10-5] FC NN
Transmission
Wavelength(nm)
through port
FDTD
DL
(F)
[5-10-20-10-5] FC NN
FDTD
DL
drop port
Transmission
Wavelength(nm)
(G)
[5-10-20-40-20-10-5] FC NN
Transmission
Wavelength(nm)
through port
FDTD
DL
(H)
[5-10-20-40-20-10-5] FC NN
FDTD
DL
drop port
Transmission
Wavelength(nm)

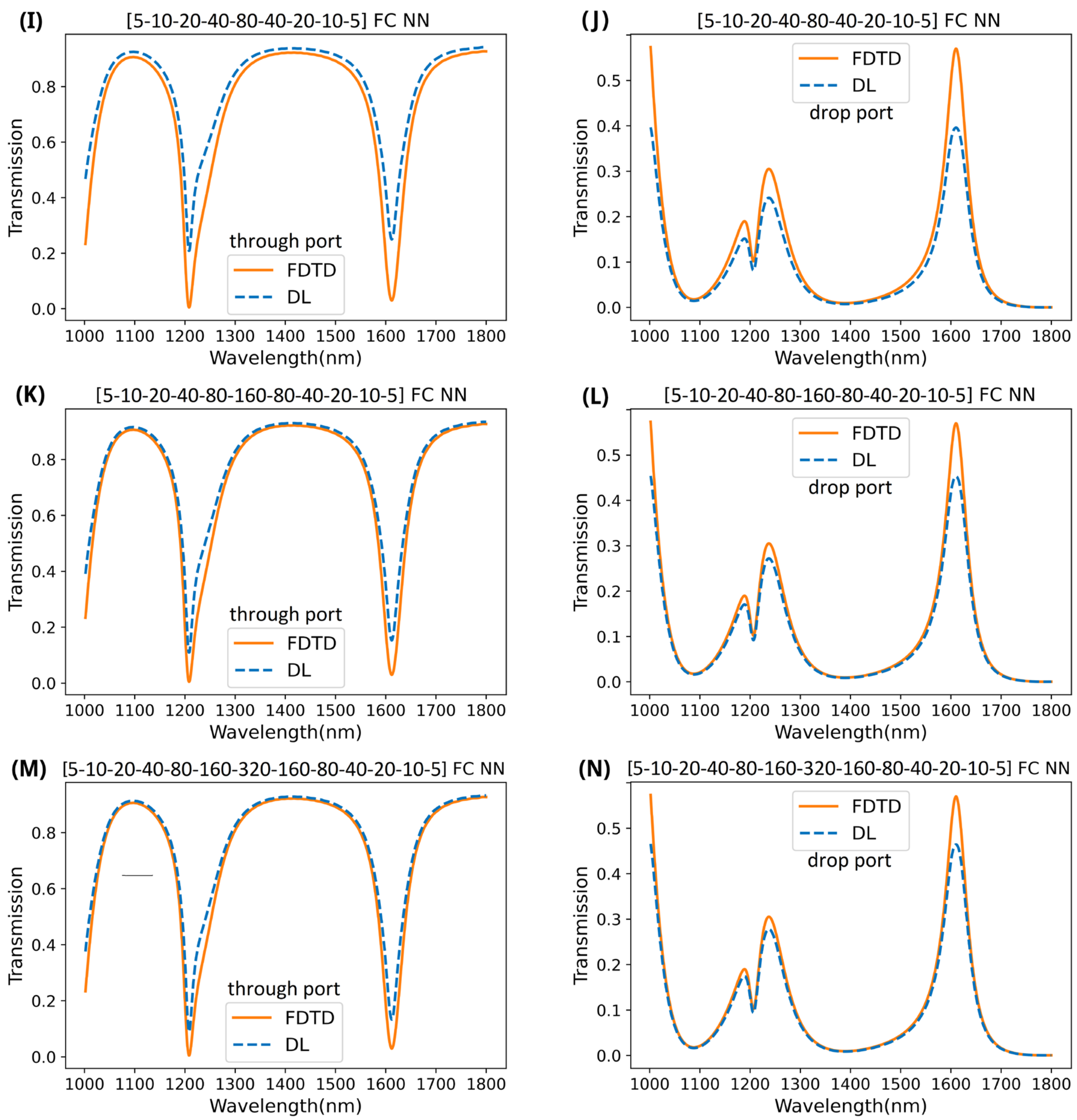

Figure S6. Evaluative comparison between DL predictions and Empirical spectra across diverse architectures: (**A**) Analysis of the through port with a single hidden layer. (**B**) Analysis of the drop port with a single hidden layer. (**C**) Analysis of the through port with three hidden layers. (**D**) Analysis of the drop port with three hidden layers. (**E**) Analysis of the through port with five hidden layers. (**F**) Analysis of the drop port with five hidden layers. (**G**) Analysis of the through port with seven hidden layers. (**H**) Analysis of the drop port with seven hidden layers. (**I**) Analysis of the through port with nine hidden layers. (**J**) Analysis of the drop port with nine hidden layers. (**K**) Analysis of the through port with 11 hidden layers. (**L**) Analysis of the drop port with 11 hidden layers. (**M**) Analysis of the through port with 13 hidden layers. (**N**) Analysis of the drop port with 13 hidden layers.

Figure S7:

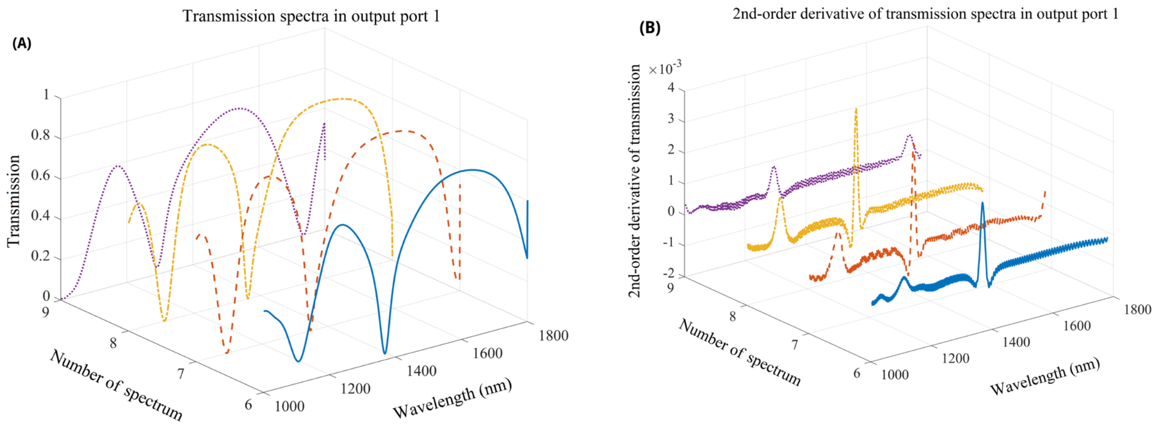

Figure S7. The relationship between dips in the spectrum and its second derivative. Elucidating the relationship between pronounced dips in the transmission spectrum and its second derivative: (**A**) displays the transmission spectrum itself, while (**B**) illustrates the corresponding second-order derivative. It is observable that an accentuated peak in the plot of the second derivative is indicative of a more defined dip in the transmission spectrum.

Figure S8:

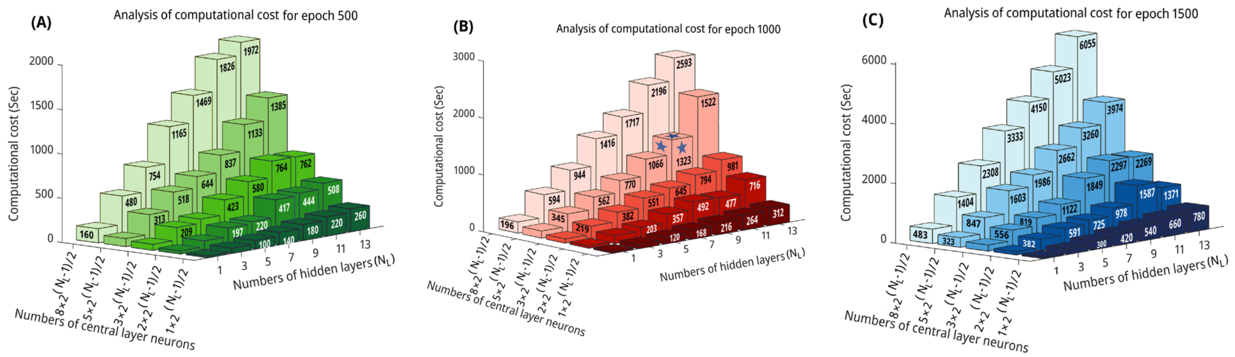

Figure S8. An examination of the computational costs. An examination of the computational expense associated with NNs featuring a variety of architectures has been performed for epochs (**A**) 500, (**B**) 1000, and (**C**) 1500. Here, the neuron count is modified by the changes in the hidden layer configuration. In such scenarios, a complex deep NN with a higher neuron count typically incurs an increased computational burden. However, a particular structure, namely one with eleven layers and 160 neurons in the central hidden layer, demonstrates that additional layers and neurons do not necessarily improve the loss metrics and might even contribute to the emergence of the vanishing gradient problem, as depicted in Figure S5 and Figure S6, while simultaneously raising computational costs.

Figure S9:

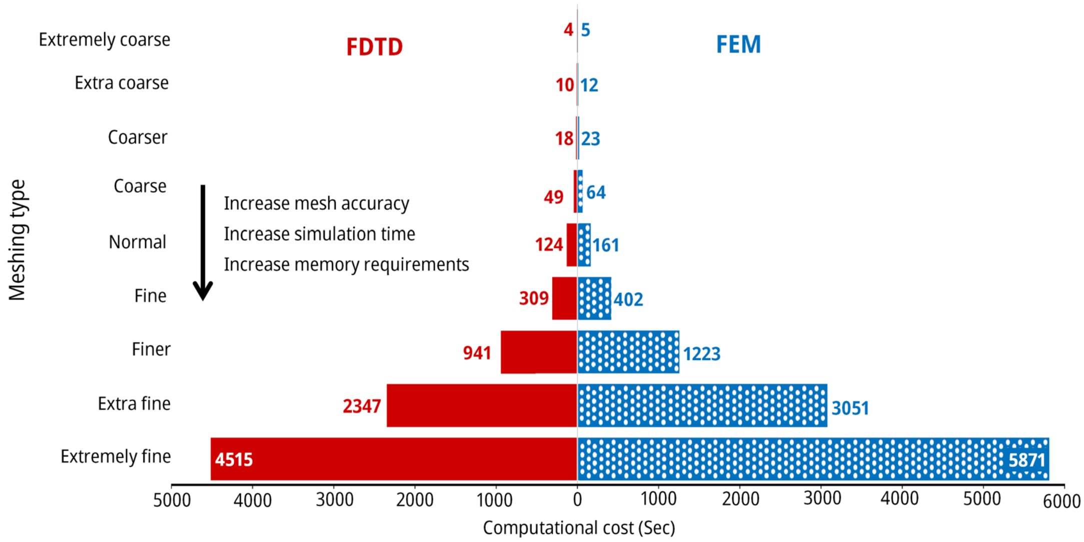

Figure S9. A comparative analysis of the computational costs. A comparative analysis has been undertaken to elucidate the computational costs incurred by the FEM and the FDTD method. When employing the most refined mesh settings, the simulations necessitated 5871 seconds for FEM and 4515 seconds for FDTD to conclude. Conversely, applying DL methodologies facilitated a profound diminution in computational time, reducing it to less than a second. This significant abridgment in the duration of simulations presents a substantial advantage, resulting in marked savings in time. This efficiency gain underscores the efficacy of DL techniques in streamlining the computational process, offering a compelling alternative to traditional numerical simulation methods.

TABLES

Table S1. Simulation values. Simulation values of the resonance characteristics of the AOPS for Taguchi experiments.

Config. No.	Structural parameters (nm)					resonance characteristics	
	W_{bus} (S_1)	W_{drop} (S_2)	W_{square} (S_3)	G_{bus} (S_4)	G_{drop} (S_5)	T_2-T_1 (%)	Lambda (nm)
1	30	30	30	15	15	71.19	1375
2	30	40	40	18	18	70.72	1672
3	30	50	50	21	21	59.34	1573
4	30	60	60	25	25	54.86	1418
5	40	30	40	21	25	58.89	1733
6	40	40	30	25	21	42.94	1349
7	40	50	60	15	18	76.39	1429
8	40	60	50	18	15	53.36	1500
9	50	30	50	25	18	47.69	1495
10	50	40	60	21	15	76.57	1438
11	50	50	30	18	25	60.5	1431
12	50	60	40	15	21	38.24	1721
13	60	30	60	15	21	32.76	1501
14	60	40	50	15	25	17.67	1580
15	60	50	40	25	15	29.76	1327
16	60	60	30	21	18	46.2	1431

Table S2. The ANOVA results. The ANOVA results provide insights into the significance of each structural factor on the contrast ratio.

Structural Parameter	Degrees of Freedom (f)	Sum of Squares (S)	Variance (V)	Pure Sum (S')	Percent P (%)
W_{bus} (S_1)	3	2437.372	812.457	2437.372	50.52
W_{drop} (S_2)	3	139.728	46.576	139.728	2.896
W_{square} (S_3)	3	555.977	185.325	555.977	11.524
G_{bus} (S_4)	3	923.871	307.957	923.871	19.149
G_{drop} (S_5)	3	767.538	255.846	767.538	15.909

Table S3. The ANOVA results. The ANOVA results provide insights into the significance of each structural factor on the resonance wavelength.

Structural Parameter	Degrees of Freedom (f)	Sum of Squares (S)	Variance (V)	Pure Sum (S')	Percent P (%)
W_{bus} (S_1)	3	8632.187	2877.395	8632.187	3.623
W_{drop} (S_2)	3	18663.687	6221.229	18663.69	7.834
W_{square} (S_3)	3	111030.682	37010.23	111030.7	46.605
G_{bus} (S_4)	3	55624.47	18541.49	55624.47	23.348
G_{drop} (S_5)	3	44281.687	14760.56	44281.69	18.587

REFERENCES

1 Nozhat, N. & Granpayeh, N. All-optical nonlinear plasmonic ring resonator switches. *Journal of Modern Optics* **61**, 1690-1695 (2014). https://doi.org/10.1080/09500340.2014.951008.

2 Ghadrdan, M. & Mansouri-Birjandi, M. A. Design and implementation of optical switches based on nonlinear plasmonic ring resonators: circular, square and octagon. *Photonics and Nanostructures-Fundamentals and Applications* **29**, 15-21 (2018). https://doi.org/10.1016/j.photonics.2018.01.003.

3 Liao, H. *et al.* Origin of third-order optical nonlinearity in Au: SiO 2 composite films on femtosecond and picosecond time scales. *Optics letters* **23**, 388-390 (1998). https://doi.org/10.1364/OL.23.000388.

4 Amiri, I. S., Afroozeh, A. & Ahmad, H. *Integrated micro-ring photonics: Principles and Applications as Slow light devices, Soliton generation and Optical transmission*. (CRC Press, 2016). https://doi.org/10.1201/9781315682990.

5 De Leon, I. & Berini, P. Amplification of long-range surface plasmons by a dipolar gain medium. *Nature Photonics* **4**, 382-387 (2010). https://doi.org/10.1038/nphoton.2010.37.

6 Liang, F., Guo, Y., Hou, S. & Quan, Q. Photonic-plasmonic hybrid single-molecule nanosensor measures the effect of fluorescent labels on DNA-protein dynamics. *Science advances* **3**, e1602991 (2017). https://doi.org/10.1126/sciadv.1602991.

7 Rodrigo, D. *et al.* Mid-infrared plasmonic biosensing with graphene. *Science* **349**, 165-168 (2015). https://doi.org/10.1126/science.aab2051.

8 Fang, Y. & Sun, M. Nanoplasmonic waveguides: towards applications in integrated nanophotonic circuits. *Light: Science & Applications* **4**, e294-e294 (2015). https://doi.org/10.1038/lsa.2015.67.

9 Barnes, W. L., Dereux, A. & Ebbesen, T. W. Surface plasmon subwavelength optics. *nature* **424**, 824-830 (2003). https://doi.org/10.1038/nature01937.

10 Kim, Y. *et al.* Ultrawideband electromagnetic metamaterial absorber utilizing coherent absorptions and surface plasmon polaritons based on double layer carbon metapatterns. *Scientific Reports* **11**, 23045 (2021). https://doi.org/10.1038/s41598-021-02303-1.

11 Li, Y., Liberal, I. & Engheta, N. Structural dispersion–based reduction of loss in epsilon-near-zero and surface plasmon polariton waves. *Science advances* **5**, eaav3764 (2019). https://doi.org/10.1126/sciadv.aav3764.

12 Wooten, F. *Optical properties of solids*. (Citeseer, 1972).
