# Supplementary material for: Nanophotonic structure inverse design for switching application using deep learning
Source: Sci Rep. 2024 Sep 10;14:21094. doi: 10.1038/s41598-024-72125-4 (PMC11387741; doi:10.1038/s41598-024-72125-4)
Supplement: Supplementary file 3 — Supplementary Legends. [file 41598_2024_72125_MOESM3_ESM.doc]

**Guide for Supplementary Video File (Animation S1):**

**Title:** The Animated Abstract.

**Legend:** The animated presentation offers a comprehensive overview of the principal achievements outlined in the article. It showcases the utilization of deep learning to realize the inverse design of a square resonator for switching.
